# Supplementary material for: Effect of price on the decision to experiment with cigarette smoking among Gambian children: a survival analysis using the Gambia 2017 Global Youth Tobacco Survey data
Source: BMJ Open. 2022 Nov 8;12(11):e061045. doi: 10.1136/bmjopen-2022-061045 (PMC9644320; doi:10.1136/bmjopen-2022-061045)
Supplement: Author's manuscript [file bmjopen-2022-061045.draft_revisions.pdf]

BMJ Open is committed to open peer review. As part of this commitment we make the peer review history of every article we publish publicly available.

When an article is published we post the peer reviewers' comments and the authors' responses online. We also post the versions of the paper that were used during peer review. These are the versions that the peer review comments apply to.

The versions of the paper that follow are the versions that were submitted during the peer review process. They are not the versions of record or the final published versions. They should not be cited or distributed as the published version of this manuscript.

BMJ Open is an open access journal and the full, final, typeset and author-corrected version of record of the manuscript is available on our site with no access controls, subscription charges or pay-per-view fees (http://bmjopen.bmj.com).

If you have any questions on BMJ Open's open peer review process please email info.bmjopen@bmj.com

BMJ Open

# The effect of price on the decision to experiment with cigarette smoking among Gambian children

| | |
|---|---|
| Journal: | *BMJ Open* |
| Manuscript ID | bmjopen-2022-061045 |
| Article Type: | Original research |
| Date Submitted by the Author: | 15-Jan-2022 |
| Complete List of Authors: | Dare, Chengetai; University of Cape Town, Research Unit on the Economics of Excisable Products (REEP), School of Economics<br>Cham, Bai; Medical Research Council Unit The Gambia at the London School of Hygiene and Tropical Medicine<br>Boachie, Micheal Kofi; University of the Witwatersrand Faculty of Health Sciences, SAMRC/Wits Centre for Health Economics and Decision Science -PRICELESS SA; University of Cape Town, Research Unit on the Economics of Excisable Products<br>Gitonga, Zachary; University of Cape Town, Research Unit on the Economics of Excisable Products (REEP), School of Economics<br>D'Alessandro, Umberto; Medical Research Council Unit The Gambia at the London School of Hygiene and Tropical Medicine<br>Walbeek, Corne; University of Cape Town, Research Unit on the Economics of Excisable Products, School of Economics |
| Keywords: | HEALTH ECONOMICS, Health policy < HEALTH SERVICES ADMINISTRATION & MANAGEMENT, Health economics < HEALTH SERVICES ADMINISTRATION & MANAGEMENT, PUBLIC HEALTH |
| | |

SCHOLARONE™
Manuscripts

*I, the Submitting Author has the right to grant and does grant on behalf of all authors of the Work (as defined in the below author licence), an exclusive licence and/or a non-exclusive licence for contributions from authors who are: i) UK Crown employees; ii) where BMJ has agreed a CC-BY licence shall apply, and/or iii) in accordance with the terms applicable for US Federal Government officers or employees acting as part of their official duties; on a worldwide, perpetual, irrevocable, royalty-free basis to BMJ Publishing Group Ltd ("BMJ") its licensees and where the relevant Journal is co-owned by BMJ to the co-owners of the Journal, to publish the Work in this journal and any other BMJ products and to exploit all rights, as set out in our licence.*

*The Submitting Author accepts and understands that any supply made under these terms is made by BMJ to the Submitting Author unless you are acting as an employee on behalf of your employer or a postgraduate student of an affiliated institution which is paying any applicable article publishing charge ("APC") for Open Access articles. Where the Submitting Author wishes to make the Work available on an Open Access basis (and intends to pay the relevant APC), the terms of reuse of such Open Access shall be governed by a Creative Commons licence – details of these licences and which Creative Commons licence will apply to this Work are set out in our licence referred to above.*

*Other than as permitted in any relevant BMJ Author's Self Archiving Policies, I confirm this Work has not been accepted for publication elsewhere, is not being considered for publication elsewhere and does not duplicate material already published. I confirm all authors consent to publication of this Work and authorise the granting of this licence.*

For peer review only

# The effect of price on the decision to experiment with cigarette smoking among Gambian children

Chengetai Dare[1] *, Cham Bai[2], Micheal K. Boachie[1,3], Zachary Gitonga[1],

Umberto D'Alessandro[2], Corné van Walbeek[1]

[1] Research Unit on the Economics of Excisable Products (REEP), School of Economics, University of Cape Town, Private Bag X3, Rondebosch 7700, South Africa

* Email: cdarejam@yahoo.com; Fax: +27 (0)21 650 4369; Mobile: +27 (0)61 880 8197

[2] Disease Control and Elimination Theme, Medical Research Council Unit The Gambia at the London School of Hygiene and Tropical Medicine, Atlantic Road, Fajara, P.O. Box 273, Banjul, The Gambia

[3] SAMRC/Wits Centre for Health Economics and Decision Science – PRICELESS SA, Wits School of Public Health, Faculty of Health Sciences, University of the Witwatersrand, Parktown, Johannesburg, 2193, South Africa.

**Keywords:** smoking experimentation, price elasticity, children, demand for cigarettes, excise tax, The Gambia

**Word count:** 2824

For peer review only

# The effect of price on the decision to experiment with cigarette smoking among Gambian children


## Abstract

### Objectives

To investigate the relationship between cigarette prices and smoking experimentation among young people in The Gambia and thereby contribute to growing the evidence base of the likely impact of excise taxes on cigarette demand in low-income and middle-income countries.

### Methods and data

We employed survival analysis to examine the effect of prices on the smoking experimentation among Gambian children. Smoking experimentation is defined as taking the first puff. The study used the 2017 Global Youth Tobacco Survey (GYTS) to obtain individual and household information on smoking behaviour and other background characteristics among students aged 12 -17 years in junior secondary schools. Price data were obtained from the Gambia Bureau of Statistics.

### Results

A 1% increase in the price of cigarettes reduces the probability to experiment with smoking by 0.7%. We also found that children are more likely to experiment with smoking if they have at least one smoking parent, friends who smoke, and see teachers who smoke. The probability to experiment with cigarette smoking increases with age and is higher in boys than in girls.

### Conclusion




For peer review only - http://bmjopen.bmj.com/site/about/guidelines.xhtml

There is strong evidence that excise taxes can play an effective role in discouraging children from experimenting with cigarette smoking. Considering the relatively low excise tax burden in the Gambia, the government should consider substantially increasing the excise tax burden.

**Strengths and limitations of this study**

- There is little evidence on the relationship between cigarette prices and tobacco use among young people in sub-Saharan Africa and this is the first study on cigarette experimentation in The Gambia.
- The study has identified key factors associated with experimenting tobacco smoking that can be used by policy makers in The Gambia and other LMICs to deter youths from smoking tobacco.
- The Global Youth Tobacco Survey is cross-sectional and focuses on students in junior secondary schools, the data may not be a good representation of Gambian youth, including those in senior secondary schools, children out of school and dropouts
- The study relied on individuals' own memory regarding past smoking behaviour and hence the dependent variable could be subject to measurement error (recall bias).

For peer review only



For peer review only - http://bmjopen.bmj.com/site/about/guidelines.xhtml

## Introduction

Smoking is the leading cause of preventable death.[1] There are over one billion smokers globally and around 80% of them live in low- and middle-income countries (LMICs).[1 2] Africa's share of the global number of smokers is rapidly increasing.[3] Over 8 million people die every year from tobacco use and/or exposure to tobacco smoke,[2 4] and 80% of all tobacco-related deaths by 2030 are predicted to occur in LMICs.[5 6] Tobacco companies are shifting their target to LMICs to build a broader consumer base, especially among young people.[7 8] In the WHO African region, the total number of smokers is projected to increase from 66 million in 2015 to 84 million by 2025;[9] this is the second-largest percentage increase after the Eastern Mediterranean region. A study that analysed data from the Global Youth Tobacco Surveys (GYTS) from 2014 to 2017 for The Gambia, Cameroon, Gabon, Comoros, Seychelles, Mauritius, Tanzania, Ghana, and Sierra Leone revealed that 75% of youths who smoked started smoking before age 15 years in all the countries.[10]

Research has shown that experimentation with cigarettes among young people is associated with future daily smoking.[11] [12]In a study among 6522 US adolescents aged 10 to 14 years, experimentation with cigarette smoking during ages 10-14 years predicted daily smoking two years later, independent of other cigarette smoking risk factors.[11] Evidence has also shown that higher prices is a deterrent of cigarette use among young people, especially in Africa. In several African countries, higher cigarette prices have contributed to a reduction of both tobacco use prevalence and intensity.[13] This has been confirmed in country-specific studies in South Africa,[14] Tanzania,[15] Kenya,[16] Zambia,[17] and Uganda.[18] Similarly, studies have found that, in Ghana and Nigeria, increased cigarette prices decreased both smoking intensity and smoking initiation.[19]

The Gambia is the smallest country on mainland Africa with a population of about 2 million. The Gambia is a low-income country and is ranked 172 out of 189 countries in the 2020 United Nations



For peer review only - http://bmjopen.bmj.com/site/about/guidelines.xhtml

Human Development Index. The country ratified the WHO Framework Convention on Tobacco Control (WHO FCTC) in September 2007. There are several regulations and policies on tobacco control in The Gambia, including the Prohibition of Smoking in Public Places Act 1998, the Tobacco Control Regulations 2019 and the Tobacco Control Act 2016. These legal instruments protect children from exposure to tobacco products. Based on these international treaties and local regulations, the country has an obligation to protect children from tobacco exposure.

The prevalence of smoking is 15.9% among Gambian adults (25-64 years old),[20] 1% among women, and 32.1% among men.[21] A 2016 survey conducted among secondary school students (12-20 years) indicated that 7.9% of boys and 1.5% of girls had smoked at least once in the past 30 days.[22] An earlier 2008 GYTS survey indicated that 10.8% of 13-15-year-old students (12.7% among boys and 8.6% among girls) had smoked at least once in the past 30 days.[23]

Like other governments across the world, The Gambian government employs tax and price measures to reduce the affordability of tobacco. For instance, in 2013 a specific excise tax was introduced on all imported tobacco products. This contributed to an increase in cigarette prices and tax revenue and to a reduction in tobacco imports.[24] However, cigarette prices remain relatively low. Data obtained from The Gambia Bureau of Statistics show that in 2019 the average retail price for a packet of 20 sticks was GMD31.70 (or US$0.62, using 2019 exchange rates), which is substantially lower than the sub-Saharan African regional average of US$1.80, and the global average price of US$3.82.[26] The tax burden (share of taxes in the retail price) was 46.3% in 2018,[26] which is below the 75% target recommended by the World Health Organisation.[27]

An increase in the excise tax is the single most important intervention to reduce smoking prevalence and intensity.[27 28] Youth are even more price-sensitive than adults, making tobacco

For peer review only



For peer review only - http://bmjopen.bmj.com/site/about/guidelines.xhtml

taxation particularly effective; this is even more so in countries with a young population.[19] Although global knowledge about the effect of prices (or taxes) as a tobacco control measure is well established, there is little evidence on the impact of prices on tobacco use experimentation among the young people in the Gambia. In this paper we fill this knowledge gap by employing survival analysis to examine the effect of prices on the decision to experiment with cigarette smoking among children in The Gambia.

## Data and methodology

We used the 2017 Global Youth Tobacco Survey (GYTS) to obtain individual and household information on smoking behaviour and other background characteristics. The GYTS is designed by the Centre for Disease Control and Prevention (CDC) as a global standard tool for monitoring tobacco use among youth and to guide the implementation and evaluation of tobacco prevention and control programmes.[29] The survey considers a cross-section of students in junior secondary schools. The 2017 GYTS covers a sample of 12,585 youths, aged 12 – 17 years. However, 1,555 respondents did not provide sufficient information about their smoking status, thereby reducing our sample size to 11,030. Smoking experimenters or ever-smokers (defined as having smoked at least once or twice over their lifetimes) was reported by 2,218 (20.1%) respondents (Table 1).

**Table 1: Demographic characteristics and smoking habits (%)**

| | N (and proportion of total sample) | Ever-smokers in the category |
|---|---|---|
| Students sampled (N=11 030) | n = 11 030 | n = 2218 (20.1%) |
| Gender (n=10 886) | | |
| Male | 44.3 | 32.9 |
| Female | 55.7 | 9.6 |
| Age (n=10 483) | | |
| 12 | 6.0 | 16.4 |
| 13 | 14.9 | 15.0 |



For peer review only - http://bmjopen.bmj.com/site/about/guidelines.xhtml

| | | |
|---|---|---|
| 14 | 23.6 | 16.7 |
| 15 | 21.7 | 18.3 |
| 16 | 17.1 | 22.4 |
| 17 | 16.7 | 28.1 |
| Parent(s) smoke(s) (n= 10 945) | 18.4 | 29.7 |
| Friend(s) smoke(s) (n=10 916) | 23.0 | 38.0 |
| Amount of pocket money (n=10 918): | | |
| Usually do not have | 12.1 | 17.4 |
| Less than GMD25 | 35.7 | 19.0 |
| GMD25-GMD50 | 32.5 | 19.8 |
| GMD51-GMD100 | 10.5 | 23.1 |
| GMD101-GMD150 | 3.9 | 23.0 |
| GMD151-GMD200 | 1.9 | 25.0 |
| Above GMD200 | 3.4 | 27.8 |
| Sees teachers smoking in school buildings | 29.7 | 25.8 |
| Regards quitting smoking as difficult (n=10 937) | 37.4 | 21.8 |
| Mean age (years) | 14.8 (SD 1.47) | 15.1 (SD 1.50) |
| Mean experimentation age (years) | | 11.4 (SD 2.96) |

The study employs a duration (or survival) model to estimate the probability of a respondent experimenting with smoking. Experimentation is defined in terms of the first time the respondent smoked (at least part of) a cigarette. It is obtained from the question: "Have you ever tried or experimented with cigarette smoking, even one or two puffs?". The timing of the transition from having never smoked into experimentation depends on the probability of experiencing a transition in period *t,* conditional on not having experienced a transition until period *t*; is also known as the hazard rate or conditional failure rate.[16] The estimation approach requires longitudinal data, making it necessary to transform the cross-sectional GYTS data into pseudo-longitudinal data.[16 19] [30]



For peer review only - http://bmjopen.bmj.com/site/about/guidelines.xhtml

As in Asare et al[19], to obtain the longitudinal data required for the duration analysis in the study of tobacco use, we retroactively inferred the year of smoking experimentation using the GYTS question on the age of smoking experimentation: "How old were you when you first tried a cigarette?". For each age, the individuals were assigned a value of 0 if they did not experiment. The person is assigned a value of 1 in the year in which they tried a cigarette and then drops out of the sample. The pseudo-longitudinal dataset was constructed on the assumption that a person is at risk of having his/her first cigarette at the age of eight.[13 23] The eldest amongst the respondents was 17 when the survey was conducted in 2017. In 2008 these respondents were eight years old. As such, our data sample excluded those with experimentation year before 2008. We also excluded respondents that reported ever-smoking, but who did not provide sufficient data on when they experimented smoking.

The pseudo-longitudinal data is then merged with the national-level cigarette price, which allows us to investigate the relationship between price and smoking experimentation. The price data was obtained from The Gambia Bureau of Statistics. The price trend is shown in Figure 1.

*Insert Figure 1 here*

For regression analysis, our baseline model uses a logistic distribution for the hazard function because of its flexibility to allow non-monotone, time-dependent changes of the hazard rate.[16 33] The regression model estimates the hazard of initiation for individual *i* in period *t* ($y_{it}$) as a function of real cigarette prices ($price_t$) and a matrix of explanatory variables ($\boldsymbol{X}_i$), which include socio-demographics (age, gender, parents' and friends' smoking status, income (pocket money) and respondents' perception on quitting smoking). Gender takes the value of 1 for female and 0 for male. Smoking status of the respondent's parents and friends takes the value of 1 if at least one of



For peer review only - http://bmjopen.bmj.com/site/about/guidelines.xhtml

the parents or friends smokes, and 0 if none of the parents or friends smoke. The respondents' income (pocket money) is derived from the question: "During an average week, how much money do you have that you can spend on yourself, however you want?". The base is 'I usually don't have', and the six other categories are 'Less than GMD25', 'GMD25-GMD50', 'GMD51-GMD100', 'GMD101-GMD150', 'GMD151-GMD200' and 'Above GMD200'. Respondents' perception on quitting smoking is derived from the GYTS question: "Once someone has started smoking tobacco, do you think it would be difficult for them to quit?" The variable is coded as 1 for "Yes" and 0 for "No". The dependent variable, i.e. cigarette smoking experimentation, is extracted from the GYTS data.[29]

The regression model is specified as:

$$y_{it} = Pr\ (initiate|no\ prior\ smoking)$$

$$= \beta_1 price_t + \beta_2 \boldsymbol{X}_{it} + \varepsilon_{it} \ldots\ldots\ldots (1)$$

We tested the robustness of the results by incorporating a discrete-time split population survival model, which relaxes the assumption that all individuals will eventually smoke. It first estimates the individual probability of ever experiencing a smoking transition, then weights the hazard function by this probability. The contribution of individual *i* to the log-likelihood function of smoking experimentation is:

$$d_i = ln\{\Pr\ (ever\ initiate) * f(t\,|\,t > 0)\} + (1 - d_i) * ln$$
$$\{Pr(ever\ initiate) + Pr(ever\ initiate) * f(t|t = 0)\} \ldots\ldots\ldots. (2)$$



For peer review only - http://bmjopen.bmj.com/site/about/guidelines.xhtml

where $d_i$ is a binary indicator for experimenting smoking at some point during the period of observation, $t$ is the time of experimentation measured in number of years since age 10, and $f(t)$ is its probability density function.

As in Vellios and Van Walbeek[30], we used the *spsurv* command for the split population estimation model. The model has been used in numerous studies.[30 34 35] The *spsurv* command uses a complementary log-log specification, which reports hazard ratios (not odds ratios) and unlike the logit specification, the response curve is asymmetric.[33] The odds ratios are calculated by taking the antilog of the coefficient. The hazard rate is calculated as (OR / (1 + OR) where OR is the odds ratio. All analyses were done with Stata v.15.

**Patients and public involvement**

Patients and the public were not involved in this study.

## Results

The regression results are shown in Table 2. The estimated odds and hazard ratios are in exponentiated form and are broadly similar. The hazard ratios are indicated in the table but are not described in this section.

**Table 2: Regression results: logistic and split population survival models**

| | Logistic regression model | | Split population model |
|---|---|---|---|
| | Coefficients | Odds ratios | Hazard ratios |
| Price elasticity of experimentation | -0.680** | 0.507** | 0.524*** |
| | (0.302) | (0.153) | (0.131) |
| Parent(s) smoke(s) | 0.371*** | 1.449*** | 1.474*** |
| | (0.082) | (0.120) | (0.093) |
| Friend(s) smoke(s) | 0.482*** | 1.620*** | 1.828*** |
| | (0.073) | (0.119) | (0.102) |
| Difficulty of quitting | 0.055 | 1.056 | 1.114* |



For peer review only - http://bmjopen.bmj.com/site/about/guidelines.xhtml

| | | | |
|---|---|---|---|
| | (0.069) | (0.073) | (0.062) |
| Teachers smoke in school buildings | 0.176** | 1.192** | 1.205*** |
| | (0.073) | (0.087) | (0.068) |
| Usually do not have pocket money | 0.000 | 1.000 | 1.000 |
| Less than GMD25 | 0.083 | 1.087 | 1.113 |
| | (0.127) | (0.138) | (0.109) |
| GMD25-GMD50 | 0.067 | 1.070 | 1.181* |
| | (0.125) | (0.134) | (0.114) |
| GMD51-GMD100 | 0.334** | 1.397** | 1.400*** |
| | (0.141) | (0.197) | (0.158) |
| GMD101-GMD150 | 0.080 | 1.084 | 1.224 |
| | (0.190) | (0.206) | (0.190) |
| GMD151-GMD200 | 0.246 | 1.279 | 1.396* |
| | (0.227) | (0.290) | (0.261) |
| Above GMD200 | 0.411** | 1.508** | 1.505*** |
| | (0.181) | (0.273) | (0.224) |
| Female | -1.403*** | 0.246*** | 1.159*** |
| | (0.083) | (0.020) | (0.016) |
| Age | 0.165*** | 1.179*** | 1.159*** |
| | (0.020) | (0.024) | (0.016) |
| Constant | -3.426*** | 0.033*** | 0.028*** |
| | (0.892) | (0.029) | (0.021) |
| Observations | 77,640 | 77,640 | 77,640 |

Standard errors in parentheses
*** p<0.01, ** p<0.05, * p<0.10

The results show that a 1% increase in the price of cigarettes reduces the probability to experiment with smoking among children by 0.7% (95% CI: -1.1 to -0.4) (as indicated in column 2).

Children with at least one smoking parent are more likely to have ever smoked a cigarette than those whose parents do not smoke (OR = 1.4, 95% CI: 1.2 to 1.7). Also, children with friends who smoke are more likely to have ever experimented with smoking a cigarette than those whose friends do not smoke (OR = 1.6, 95% CI: 0.4 to 1.9).

Children who see their teachers smoking inside school buildings are more likely to have ever experimented with smoking a cigarette than those who do not see teachers smoking inside school



For peer review only - http://bmjopen.bmj.com/site/about/guidelines.xhtml

For peer review only

buildings (OR = 1.2, 95% CI: 1.0 to 1.4). Income (i.e., having access to pocket money) did not have a clear effect on the decision to experiment with smoking.

Female youths are less likely to have ever smoked a cigarette than male youths (OR = 0.2, 95% CI: 0.2 to 0.3). The results also show that a one-year increase in age increases the probability to experiment with smoking (OR = 1.2, 95% CI:1.1 to 1.2). Figure 2 show the smoking initiation hazard rates by age and gender. The hazard rates are derived from the regression analyses.

*Insert Figure 2 here*

Figure 2 shows a positive correlation between age and the probability to experiment with smoking cigarettes among the youth in The Gambia. The probability to experiment smoking is higher among boys than among girls.

## Discussion

The price elasticity of demand for smoking experimentation is estimated to be -0.7 among children in The Gambia. This implies that a 10% rise in the price of cigarettes is associated with a 7% reduction in the probability to experiment smoking among the Gambian children. Increasing excise taxes could be a highly effective tobacco-control tool in The Gambia. The excise tax typically increases the retail price of cigarettes, thereby reducing the propensity to experiment smoking. Considering that the current tax burden of 46.3% is well below the 75% target recommended by the World Health Organisation,[36] there is room for the government to further raise the excise tax on tobacco. Raising tobacco tax is a 'win-win' policy, as it generates extra revenue for the government while discouraging tobacco consumption through higher prices, thereby improving public health.[24]



For peer review only - http://bmjopen.bmj.com/site/about/guidelines.xhtml

The study established that children with smoking friends were more likely to have ever smoked a cigarette than those whose friends do not smoke. Similarly, children from households with at least one parent who currently smokes were more likely to have ever smoked a cigarette. This could be because, as children are more exposed to smoking (especially by parents) they would regard smoking as normal. Also, if the parents smoke, access to cigarettes is easier, than if the parents do not smoke. The results from this study are in line with the findings of Jallow et al[37] who found that children in smoking families were more likely to smoke than those in non-smoking families. We also found that children are more likely to experiment with cigarette smoking if they are see their teachers smoking in school buildings. Nevertheless, our study found that peer influence was more important in experimenting with smoking than both parental and teachers' influence. A systematic review of studies conducted among high school and university students in Ethiopia and other studies in the US found that peer pressure had a significant influence on young peoples' initiation and smoking of cigarettes.[28 38] Increases in the price will have a direct and indirect influence on smoking. The direct influence will be through the price elasticity of demand. The indirect influence will be through the peers; if the price increases, the peers will smoke less (or not at all), thus decreasing the peer pressure.

The Gambian youths start smoking as early as at age eight a result similar to a previously published study.[21] The likelihood of smoking increases exponentially. As in many other countries, the probability of smoking is higher among boys than among girls. This is mirrored by the smoking prevalence in the general population, where smoking prevalence among women is very low (about 1%), while it is much higher for men (32.1%).[21] However, the smoking rate among girls is substantially higher than among women, suggesting that female smoking prevalence may increase sharply in future, as girls step into adulthood. Based on the findings from this study, the Gambian



For peer review only - http://bmjopen.bmj.com/site/about/guidelines.xhtml

government should consider substantially increasing cigarette taxes to discourage youths from smoking.

Although the estimates presented in this study are useful guides for devising suitable tobacco-tax policy measures, these findings have limitations. For instance, the study relied on individuals' own memory regarding past smoking behaviour. As such, the dependent variable could be subject to measurement error with an unknown bias. However, this challenge is more prevalent among older respondents. Considering that our sample was composed of children, we believe that our findings are robust. Another important limitation is that the study relied on a pseudo-panel where several individual demographic and socio-economic characteristics were fixed at the time of the survey, and therefore can only be interpreted in a cross-sectional setting. Nevertheless, the fact that the estimated effects of these covariates were generally in the expected directions and the fact that the logit and split population analyses generated similar estimates enhanced our confidence in the obtained results. Also, considering that the 2017 GYTS is a cross-sectional survey among students in junior secondary schools, the data may not be a good representation of Gambian youth, e.g. those in senior secondary schools and school dropouts. We do not have information on what happens beyond this age group, because the data are not collected. In addition, some important information such as the respondents' region, race, and religion were not collected. Had they been available, these variables could have been used in the regression analysis.

## Conclusion

We found the price elasticity of demand for smoking experimentation to be -0.7 among children who have smoked at least once or twice in their lives. Our results indicate that excise taxes can play an effective role in discouraging children from experimenting with smoking. Considering the



For peer review only - http://bmjopen.bmj.com/site/about/guidelines.xhtml

current relatively low excise tax burden, the government should consider increasing the excise tax burden in line with the recommendations of the World Health Organisation.[27]

**Acknowledgements:** Special thanks to Nicole Vellios who reviewed earlier drafts.

**Contributors:** CD and MKB conceptualised the study and conducted the data analysis. CD, MKB and CB wrote the first draft of the manuscript. CvW, ZG and UD'A contributed to the drafting and revision of the manuscript.

**Funding:** This study was primarily supported by the UK Research and Innovation (UKRI) with funding from the Global Challenges Research Fund (MR/P027946/2), as part of the Tobacco Control Capacity Programme (TCCP). The TCCP is a programme of capacity development and research co-ordinated by the University of Edinburgh, Scotland and involves 15 partner institutions from Africa, South Asia and the United Kingdom. The study was also supported by the South African Medical Research Council (grant no: 23108).

**Competing interests:** None.

**Patient consent for publication:** Not required.

**Ethics approval:** N/A. The data is publicly available.

**Data sharing statement:** Data are publicly available on:

https://nccd.cdc.gov/GTSSDataSurveyResources/Ancillary/DataReports.aspx?CAID=2 .

## References

1. National Cancer Institute and World Health Organization. The Economics of Tobacco and Tobacco Control: National Cancer Institute Tobacco Control Monograph 21 NIH



For peer review only - http://bmjopen.bmj.com/site/about/guidelines.xhtml

Publication No 16-CA-8029A: Bethesda, MD: U.S. Department of Health and Human Services, National Institutes of Health, National Cancer Institute; and Geneva, CH: World Health Organization, 2016.

2. World Health Organization. Global Health Observatory data: prevalence of tobacco smoking. *Web address: http://www who int/gho/tobacco/use/en/, Accessed date* 2020;28

3. Bilano V, Gilmour S, Moffiet T, et al. Global trends and projections for tobacco use, 1990–2025: an analysis of smoking indicators from the WHO Comprehensive Information Systems for Tobacco Control. *The Lancet* 2015;385(9972):966-76.

4. World health Organization. Tobacco Factsheet 2020 [Available from: https://www.who.int/en/news-room/fact-sheets/detail/tobacco [Accessed 02 March 2021].

5. Forouzanfar MH, Afshin A, Alexander LT, et al. Global, regional, and national comparative risk assessment of 79 behavioural, environmental and occupational, and metabolic risks or clusters of risks, 1990–2015: a systematic analysis for the Global Burden of Disease Study 2015. *The lancet* 2016;388(10053):1659-724.

6. Blecher EH, Ross H. Tobacco use in Africa: tobacco control through prevention. *American Cancer Society* 2015 doi: 10.13140/RG.2.1.1038.2247

7. Chandora R, Song Y, Chaussard M, et al. Youth access to cigarettes in six sub-Saharan African countries. *Preventive Medicine* 2016;91:S23-S27.

8. Organization WH. WHO report on the global tobacco epidemic, 2013: enforcing bans on tobacco advertising, promotion and sponsorship: World Health Organization 2013.

9. World Health Organization. WHO global report on trends in prevalence of tobacco smoking 2000-2025: World Health Organization 2018.



For peer review only - http://bmjopen.bmj.com/site/about/guidelines.xhtml

10. Chido-Amajuoyi OG, Fueta P, Mantey D. Age at Smoking Initiation and Prevalence of Cigarette Use Among Youths in Sub-Saharan Africa, 2014-2017. *JAMA network open* 2021;4(5):e218060-e60.

11. Sargent JD, Gabrielli J, Budney A, et al. Adolescent smoking experimentation as a predictor of daily cigarette smoking. *Drug Alcohol Depend* 2017;175:55-59. doi: 10.1016/j.drugalcdep.2017.01.038 [published Online First: 2017/04/10]

12. Friedman AS, Buckell J, Sindelar JL. Patterns of Youth Cigarette Experimentation and Onset of Habitual Smoking. *Am J Prev Med* 2019;56(6):803-10. doi: 10.1016/j.amepre.2019.02.009 [published Online First: 2019/04/22]

13. Ho L-M, Schafferer C, Lee J-M, et al. The effect of cigarette price increases on cigarette consumption, tax revenue, and smoking-related death in Africa from 1999 to 2013. *International journal of public health* 2017;62(8):899-909.

14. Mukong AK, Tingum EN. The demand for cigarettes: New evidence from South Africa. *Development Southern Africa* 2020;37(1):40-54.

15. Kidane A, Mduma J, Naho A, et al. The demand for cigarettes in tanzania and implications for tobacco taxation policy. *Advances in economics and business* 2015;3(10):428.

16. Dauchy E, Ross H. The effect of price and tax policies on the decision to smoke among men in Kenya. *Addiction* 2019;114(7):1249-63.

17. Stoklosa M, Goma F, Nargis N, et al. Price, tax and tobacco product substitution in Zambia: findings from the ITC Zambia Surveys. *Tobacco Control* 2019;28(Suppl 1):s45-s52.

18. Chelwa G, Van Walbeek C. Does cigarette demand respond to price increases in Uganda? Price elasticity estimates using the Uganda National Panel Survey and Deaton's method. *BMJ open* 2019;9(3):e026150.



For peer review only - http://bmjopen.bmj.com/site/about/guidelines.xhtml

19. Asare S, Stoklosa M, Drope J, et al. Effects of Prices on Youth Cigarette Smoking and Tobacco Use Initiation in Ghana and Nigeria. *International journal of environmental research and public health* 2019;16(17):3114.

20. Cham B, Scholes S, Ng Fat L, et al. Burden of hypertension in The Gambia: evidence from a national World Health Organization (WHO) STEP survey. *International journal of epidemiology* 2018;47(3):860-71.

21. Cham B, Scholes S, Groce NE, et al. Prevalence and predictors of smoking among Gambian men: a cross-sectional national WHO STEP survey. *International journal of environmental research and public health* 2019;16(23):4719.

22. Jallow IK, Britton J, Langley T. Prevalence and determinants of tobacco use among young people in The Gambia. *BMJ global health* 2017;2(4)

23. Manneh E, Gambia. A Global Youth Tobacco Survey (GYTS) Country Report 2008. *Government of The Gambia: Banjul, The Gambia* 2008

24. Nargis N, Manneh Y, Krubally B, et al. How effective has tobacco tax increase been in the Gambia? A case study of tobacco control. *BMJ open* 2016;6(8):e010413.

25. Chisha Z, Janneh ML, Ross H. Consumption of legal and illegal cigarettes in the Gambia. *Tobacco control* 2020;29(Suppl 4):s254-s59.

26. World Health Organization. WHO report on the global tobacco epidemic 2019: Offer help to quit tobacco use. Geneva: World Health Organization, 2019.

27. World Health Organization. Taxation 2020 [Available from: https://www.euro.who.int/en/health-topics/disease-prevention/tobacco/publications/key-policy-documents/who-framework-convention-on-tobacco-control-who-fctc/key-areas-of-tobacco-control-policy/taxation Access: 20 July 2020.



For peer review only - http://bmjopen.bmj.com/site/about/guidelines.xhtml

28. IARC. IARC handbooks of cancer prevention, tobacco control, vol. 14: effectiveness of tax and price policies for tobacco control. *Lyon, France* 2011

29. Centers for Disease Control and Prevention. Global Tobacco Surveillance System Data. 2021 [Available from: https://nccd.cdc.gov/GTSSDataSurveyResources/Ancillary/DataReports.aspx?CAID=2 [Accessed: 23 January 2021].

30. Vellios N, van Walbeek C. Determinants of regular smoking onset in South Africa using duration analysis. *BMJ open* 2016;6(7)

31. Marti J. The impact of tobacco control expenditures on smoking initiation and cessation. *Health economics* 2014;23(12):1397-410.

32. Madden D. Tobacco taxes and starting and quitting smoking: does the effect differ by education? *Applied Economics* 2007;39(5):613-27.

33. Lambert PC. Modeling of the cure fraction in survival studies. *The Stata Journal* 2007;7(3):351-75.

34. Lillard DR, Molloy E, Sfekas A. Smoking initiation and the iron law of demand. *Journal of health economics* 2013;32(1):114-27.

35. Kidd MP, Hopkins S. The hazards of starting and quitting smoking: some Australian evidence. *Economic Record* 2004;80(249):177-92.

36. World Health Organization. Taxation 2021 [Available from: https://www.euro.who.int/en/health-topics/disease-prevention/tobacco/publications/key-policy-documents/who-framework-convention-on-tobacco-control-who-fctc/key-areas-of-tobacco-control-policy/taxation accessed 06 June 2021.



For peer review only - http://bmjopen.bmj.com/site/about/guidelines.xhtml

37. Jallow IK, Britton J, Langley T. Prevalence and determinants of susceptibility to tobacco smoking among students in the Gambia. *Nicotine and Tobacco Research* 2019;21(8):1113-21.

38. Leshargie CT, Alebel A, Kibret GD, et al. The impact of peer pressure on cigarette smoking among high school and university students in Ethiopia: A systemic review and meta-analysis. *PloS one* 2019;14(10):e0222572.

For peer review only

**Figure 1: Average real price, 2008 – 2017**

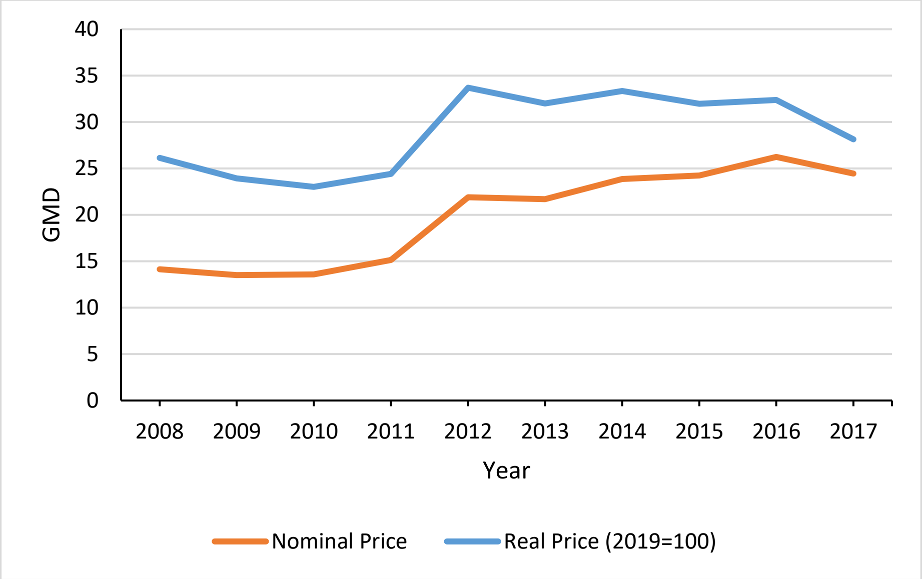


**Source:** The Gambia Bureau of Statistics

For peer review only

**Figure 2: Smoking hazard rates for smoking experimentation**

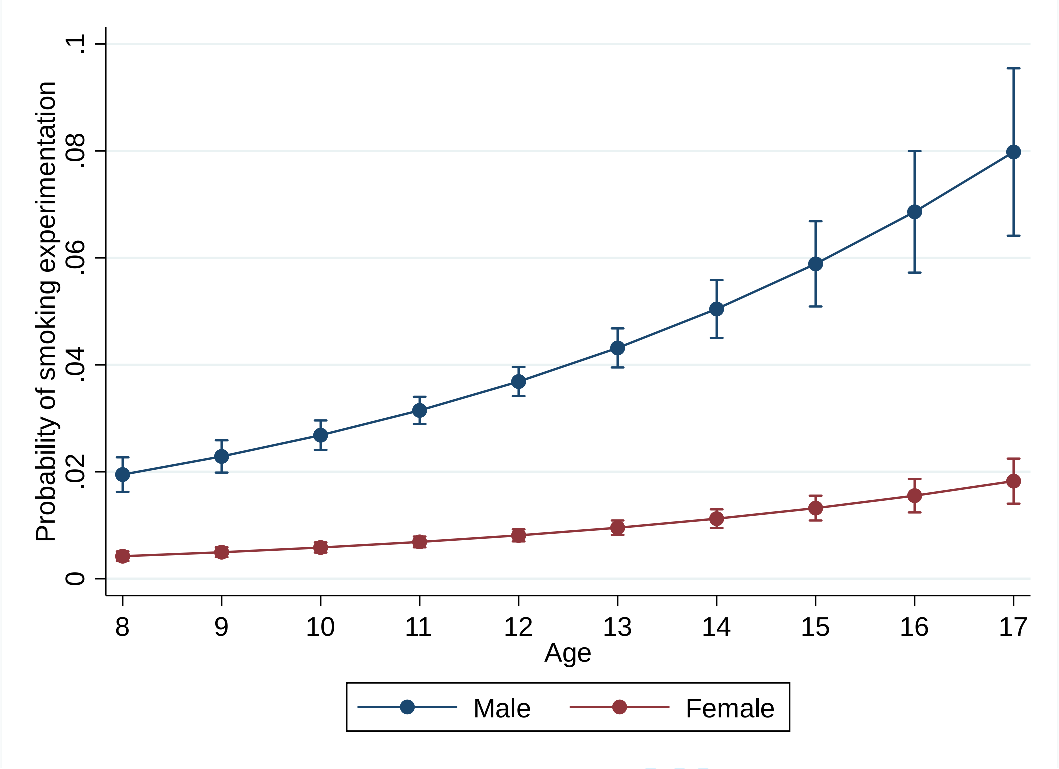


ew only

STROBE Statement—Checklist of items that should be included in reports of ***cross-sectional studies***

| | Item No | Recommendation | Page No |
|---|---|---|---|
| **Title and abstract** | 1 | (*a*) Indicate the study's design with a commonly used term in the title or the abstract | 2 |
| | | (*b*) Provide in the abstract an informative and balanced summary of what was done and what was found | 3 |
| **Introduction** | | | |
| Background/rationale | 2 | Explain the scientific background and rationale for the investigation being reported | 4-6 |
| Objectives | 3 | State specific objectives, including any prespecified hypotheses | 6 |
| **Methods** | | | |
| Study design | 4 | Present key elements of study design early in the paper | 7 |
| Setting | 5 | Describe the setting, locations, and relevant dates, including periods of recruitment, exposure, follow-up, and data collection | 7-8 |
| Participants | 6 | (*a*) Give the eligibility criteria, and the sources and methods of selection of participants | 8 |
| Variables | 7 | Clearly define all outcomes, exposures, predictors, potential confounders, and effect modifiers. Give diagnostic criteria, if applicable | 8-9 |
| Data sources/ measurement | 8* | For each variable of interest, give sources of data and details of methods of assessment (measurement). Describe comparability of assessment methods if there is more than one group | 8-9 |
| Bias | 9 | Describe any efforts to address potential sources of bias | 10 |
| Study size | 10 | Explain how the study size was arrived at | 8 |
| Quantitative variables | 11 | Explain how quantitative variables were handled in the analyses. If applicable, describe which groupings were chosen and why | 9 |
| Statistical methods | 12 | (*a*) Describe all statistical methods, including those used to control for confounding | 9-10 |
| | | (*b*) Describe any methods used to examine subgroups and interactions | 9-10 |
| | | (*c*) Explain how missing data were addressed | 8 |
| | | (*d*) If applicable, describe analytical methods taking account of sampling strategy | 7-8 |
| | | (*e*) Describe any sensitivity analyses | 9 |
| **Results** | | | |
| Participants | 13* | (a) Report numbers of individuals at each stage of study—eg numbers potentially eligible, examined for eligibility, confirmed eligible, included in the study, completing follow-up, and analysed | 8 |
| | | (b) Give reasons for non-participation at each stage | |
| | | (c) Consider use of a flow diagram | |
| Descriptive data | 14* | (a) Give characteristics of study participants (eg demographic, clinical, social) and information on exposures and potential confounders | 7 |
| | | (b) Indicate number of participants with missing data for each variable of interest | |
| Outcome data | 15* | Report numbers of outcome events or summary measures | |
| Main results | 16 | (*a*) Give unadjusted estimates and, if applicable, confounder-adjusted estimates and their precision (eg, 95% confidence interval). Make clear which confounders were adjusted for and why they were included | 11-12 |



For peer review only - http://bmjopen.bmj.com/site/about/guidelines.xhtml

| | | | |
|---|---|---|---|
| | | (*b*) Report category boundaries when continuous variables were categorized | |
| | | (*c*) If relevant, consider translating estimates of relative risk into absolute risk for a meaningful time period | |
| Other analyses | 17 | Report other analyses done—eg analyses of subgroups and interactions, and sensitivity analyses | |
| **Discussion** | | | |
| Key results | 18 | Summarise key results with reference to study objectives | 10-12 |
| Limitations | 19 | Discuss limitations of the study, taking into account sources of potential bias or imprecision. Discuss both direction and magnitude of any potential bias | 14 |
| Interpretation | 20 | Give a cautious overall interpretation of results considering objectives, limitations, multiplicity of analyses, results from similar studies, and other relevant evidence | 12-13 |
| Generalisability | 21 | Discuss the generalisability (external validity) of the study results | 14 |
| **Other information** | | | |
| Funding | 22 | Give the source of funding and the role of the funders for the present study and, if applicable, for the original study on which the present article is based | 15 |

*Give information separately for exposed and unexposed groups.

**Note:** An Explanation and Elaboration article discusses each checklist item and gives methodological background and published examples of transparent reporting. The STROBE checklist is best used in conjunction with this article (freely available on the Web sites of PLoS Medicine at http://www.plosmedicine.org/, Annals of Internal Medicine at http://www.annals.org/, and Epidemiology at http://www.epidem.com/). Information on the STROBE Initiative is available at www.strobe-statement.org.

For peer review only

BMJ Open

# The effect of price on the decision to experiment with cigarette smoking among Gambian children: A survival analysis using the Gambia 2017 Global Youth Tobacco Survey data



| | |
|---|---|
| Journal: | *BMJ Open* |
| Manuscript ID | bmjopen-2022-061045.R1 |
| Article Type: | Original research |
| Date Submitted by the Author: | 06-Jul-2022 |
| Complete List of Authors: | Dare, Chengetai; University of Cape Town, Research Unit on the Economics of Excisable Products (REEP), School of Economics<br>Cham, Bai; Medical Research Council Unit The Gambia at the London School of Hygiene and Tropical Medicine<br>Boachie, Micheal Kofi; University of the Witwatersrand Faculty of Health Sciences, SAMRC/Wits Centre for Health Economics and Decision Science -PRICELESS SA; University of Cape Town, Research Unit on the Economics of Excisable Products<br>Gitonga, Zachary; University of Cape Town, Research Unit on the Economics of Excisable Products (REEP), School of Economics<br>D'Alessandro, Umberto; Medical Research Council Unit The Gambia at the London School of Hygiene and Tropical Medicine<br>Walbeek, Corne; University of Cape Town, Research Unit on the Economics of Excisable Products, School of Economics |
| <b>Primary Subject Heading</b>: | Smoking and tobacco |
| Secondary Subject Heading: | Public health, Health policy, Health economics |
| Keywords: | HEALTH ECONOMICS, Health policy < HEALTH SERVICES ADMINISTRATION & MANAGEMENT, Health economics < HEALTH SERVICES ADMINISTRATION & MANAGEMENT, PUBLIC HEALTH |
| | |



SCHOLARONE™
Manuscripts

*I, the Submitting Author has the right to grant and does grant on behalf of all authors of the Work (as defined in the below author licence), an exclusive licence and/or a non-exclusive licence for contributions from authors who are: i) UK Crown employees; ii) where BMJ has agreed a CC-BY licence shall apply, and/or iii) in accordance with the terms applicable for US Federal Government officers or employees acting as part of their official duties; on a worldwide, perpetual, irrevocable, royalty-free basis to BMJ Publishing Group Ltd ("BMJ") its licensees and where the relevant Journal is co-owned by BMJ to the co-owners of the Journal, to publish the Work in this journal and any other BMJ products and to exploit all rights, as set out in our licence.*

*The Submitting Author accepts and understands that any supply made under these terms is made by BMJ to the Submitting Author unless you are acting as an employee on behalf of your employer or a postgraduate student of an affiliated institution which is paying any applicable article publishing charge ("APC") for Open Access articles. Where the Submitting Author wishes to make the Work available on an Open Access basis (and intends to pay the relevant APC), the terms of reuse of such Open Access shall be governed by a Creative Commons licence – details of these licences and which Creative Commons licence will apply to this Work are set out in our licence referred to above.*

*Other than as permitted in any relevant BMJ Author's Self Archiving Policies, I confirm this Work has not been accepted for publication elsewhere, is not being considered for publication elsewhere and does not duplicate material already published. I confirm all authors consent to publication of this Work and authorise the granting of this licence.*

For peer review only

# The effect of price on the decision to experiment with cigarette smoking among Gambian children: A survival analysis using the Gambia 2017 Global Youth Tobacco Survey data

Chengetai Dare[1 *], Cham Bai[2], Micheal K. Boachie[1,3], Zachary Gitonga[1], Umberto D'Alessandro[2], Corné van Walbeek[1]

[1] Research Unit on the Economics of Excisable Products (REEP), School of Economics, University of Cape Town, Private Bag X3, Rondebosch 7700, South Africa

[*] Email: cdarejam@yahoo.com; Fax: +27 (0)21 650 4369; Mobile: +27 (0)61 880 8197

[2] Disease Control and Elimination Theme, Medical Research Council Unit The Gambia at the London School of Hygiene and Tropical Medicine, Atlantic Road, Fajara, P.O. Box 273, Banjul, The Gambia

[3] SAMRC/Wits Centre for Health Economics and Decision Science – PRICELESS SA, Wits School of Public Health, Faculty of Health Sciences, University of the Witwatersrand, Parktown, Johannesburg, 2193, South Africa.

**Keywords:** smoking experimentation, price elasticity, children, demand for cigarettes, excise tax, The Gambia

**Word count:** 3215

For peer review only



For peer review only - http://bmjopen.bmj.com/site/about/guidelines.xhtml

# The effect of price on the decision to experiment with cigarette smoking among Gambian children: A survival analysis using the Gambia 2017 Global Youth Tobacco Survey data


## Abstract

**Objectives:** To investigate the relationship between cigarette prices and smoking experimentation among children in The Gambia, and thereby expanding the evidence base of the likely impact of excise taxes on cigarette demand in low-income and middle-income countries.

**Design:** A survival analysis using the Gambia 2017 Global Youth Tobacco Survey data.

**Setting:** The Gambia.

**Participants:** The survey sample was 12,585 youths, aged 12 – 17 years but our analysis was restricted to 11,030 respondents with information on smoking status.

**Primary and secondary outcome measures:** Our outcome variable was smoking experimentation defined as the first time the respondent smoked (at least part of) a cigarette.

**Results:** A 1% increase in the price of cigarettes reduces the probability to experiment with smoking by 0.7%. We also found that children are more likely to experiment with smoking if they have at least one smoking parent, friends who smoke, and see teachers who smoke. The probability to experiment with cigarette smoking increases with age and is higher among boys than girls.

**Conclusion:** There is strong evidence that increasing excise taxes can play an effective role in discouraging children from experimenting with cigarette smoking. Considering the relatively low excise tax burden in the Gambia, the government should consider substantially increasing the excise tax burden.


**Strengths and limitations of this study**



For peer review only - http://bmjopen.bmj.com/site/about/guidelines.xhtml

- Survival analysis using pseudo-longitudinal data (obtained from cross sectional data) is a good strategy in estimating longitudinal outcomes in settings where longitudinal studies are costly and/or difficult to conduct.
- There are very few tobacco demand studies in Sub-Saharan Africa that employed survival analysis, and this is the first in the Gambia.
- The Global Youth Tobacco Survey is cross-sectional and focuses on students in junior secondary schools, the data may not be a good representation of Gambian youth, including those in senior secondary schools, children out of school and school dropouts.
- The study relied on individuals' own memory regarding past smoking behaviour and hence the dependent variable could be subject to measurement error (specifically, recall bias).

For peer review only

## Introduction

Smoking is the leading cause of preventable death.[1] There are over one billion smokers globally and around 80% of them live in low- and middle-income countries (LMICs).[1, 2] Africa's share of the global number of smokers is rapidly increasing.[3] Over 8 million people die every year from tobacco use and/or exposure to tobacco smoke,[2, 4] and 80% of all tobacco-related deaths by 2030 are predicted to occur in LMICs.[5, 6] Tobacco companies are shifting their target to LMICs to build a broader consumer base, especially among young people.[7, 8] In the WHO African region, the total number of smokers is projected to increase by 27.3% from 66 million in 2015 to 84 million by 2025;[9] this is the second-largest percentage increase after the Eastern Mediterranean region.

Research has shown that experimentation with cigarettes among young people is associated with future daily smoking.[10, 11] In a study among 6522 US adolescents aged 10 to 14 years, experimentation with cigarette smoking during ages 10-14 years predicted daily smoking two years later, independent of other cigarette smoking risk factors.[10] Evidence has also shown that higher prices is a deterrent of cigarette use among young people, especially in Africa. In several African countries, higher cigarette prices have contributed to a reduction of both tobacco use prevalence and intensity.[12] This has been confirmed in country-specific studies in Sierra Leone,[13] South Africa,[14] Tanzania,[15] Kenya,[16] Zambia,[17] and Uganda.[18] Similarly, studies have found that, in Ghana and Nigeria, increased cigarette prices decreased both smoking intensity and smoking initiation.[19]

The Gambia is the smallest country on mainland Africa with a population of about 2 million. It is a low-income country and is ranked 172 out of 189 countries in the 2020 United Nations Human Development Index,[20] with per capita GDP of $773.[21] The country ratified the WHO



For peer review only - http://bmjopen.bmj.com/site/about/guidelines.xhtml

Framework Convention on Tobacco Control (WHO FCTC) in September 2007.[22] There are several regulations and policies on tobacco control in The Gambia, including the Prohibition of Smoking in Public Places Act 1998, the Tax Policy Reform Act 2013, the Tobacco Control Act 2016 and the Tobacco Control Regulations 2019.[23, 24] These legal instruments seek to protect children from exposure to tobacco products. However, despite the numerous achievements in tobacco control as highlighted above, there are still gaps, especially on the implementation of smoke-free regulation in The Gambia.[24] There has also been some regression, specifically with the Tax Policy Reform Act, which has been stalled since 2017, following a change in government.

The prevalence of smoking is 15.9% among Gambian adults (25-64 years old),[25] 1% among women, and 32.1% among men.[26] A 2016 survey conducted among secondary school students (12-20 years) indicated that 7.9% of boys and 1.5% of girls had smoked at least once in the past 30 days.[27] An earlier 2008 GYTS survey indicated that 10.8% of 13-15-year-old students (12.7% among boys and 8.6% among girls) had smoked at least once in the past 30 days.[28]

Like other governments across the world, The Gambian government employs tax and price measures to reduce the affordability of tobacco. For instance, in 2013 a specific excise tax was introduced on all imported tobacco products. This contributed to an increase in cigarette prices and tax revenue and to a reduction in tobacco imports.[29] However, cigarette prices remain relatively low. Data obtained from The Gambia Bureau of Statistics[30] show that the average retail price for a packet of 20 sticks (of the most popular brands) ranged between GMD26.15 (US$0.51) and GMD31.70 (or US$0.62, using 2019 exchange rates) between 2008 and 2019, which is substantially lower than the sub-Saharan African regional average of US$1.80, and the global average price of US$3.82.[31] The tax burden (share of taxes in the retail price) was 46.3% in 2018,[31] which is below the 75% target recommended by the World Health Organisation.[32]



For peer review only - http://bmjopen.bmj.com/site/about/guidelines.xhtml

An increase in the excise tax is the single most important intervention to reduce smoking prevalence and intensity.[32, 33] Youth are even more price-sensitive than adults, making tobacco taxation particularly effective; this is even more so in countries with a young population.[19] Although global knowledge about the effect of prices (or taxes) as a tobacco control measure is well established, according to our knowledge, there is no evidence on the impact of prices on tobacco use experimentation among the children in the Gambia. In this paper we fill this knowledge gap by employing survival analysis to examine the effect of prices on the decision to experiment with cigarette smoking among children in The Gambia.

## Data and methodology

We used the 2017 Global Youth Tobacco Survey (GYTS)[34] to obtain individual and household information on smoking behaviour and other background characteristics. The GYTS is a nationally representative school-based survey designed by the Centre for Disease Control and Prevention (CDC) as a global standard tool for monitoring tobacco use among youth and to guide the implementation and evaluation of tobacco prevention and control programmes.[34] The survey considers a cross-section of students in junior secondary schools (Grades 7-9). It does not follow individuals over time, but provides a snapshot on their smoking patterns. The sample is drawn using a two-stage cluster-sampling design.[35, 36] Schools are selected with probability proportional to school enrolment size during the first stage, and then classes within participating schools are selected as a systematic equal probability sample with a random start during the second stage.[36] All students in the selected classes are eligible to participate in the survey.[34, 36]

The 2017 GYTS covers a sample of 12,585 youths, aged 12 – 17 years. However, 1,555 respondents did not provide sufficient information about their smoking status, thereby reducing



For peer review only - http://bmjopen.bmj.com/site/about/guidelines.xhtml

our sample size to 11,030. Smoking experimenters or ever-smokers (defined as having smoked at least once or twice over their lifetimes) was reported by 2,218 (20.1%) respondents (Table 1).

**Table 1: Demographic characteristics and smoking habits (%)**

| | N (and proportion of total sample) | Ever-smokers in the category |
|---|---|---|
| Students sampled (N=11 030) | n = 11 030 | n = 2218 (20.1%) |
| Gender (n=10 886) | | |
| Male | 44.3 | 32.9 |
| Female | 55.7 | 9.6 |
| Age (n=10 483) | | |
| 12 | 6.0 | 16.4 |
| 13 | 14.9 | 15.0 |
| 14 | 23.6 | 16.7 |
| 15 | 21.7 | 18.3 |
| 16 | 17.1 | 22.4 |
| 17 | 16.7 | 28.1 |
| Parent(s) smoke(s) (n= 10 945) | 18.4 | 29.7 |
| Friend(s) smoke(s) (n=10 916) | 23.0 | 38.0 |
| Amount of pocket money per week (n=10 918): | | |
| Usually do not have | 12.1 | 17.4 |
| Less than GMD25 | 35.7 | 19.0 |
| GMD25-GMD50 | 32.5 | 19.8 |
| GMD51-GMD100 | 10.5 | 23.1 |
| GMD101-GMD150 | 3.9 | 23.0 |
| GMD151-GMD200 | 1.9 | 25.0 |
| Above GMD200 | 3.4 | 27.8 |
| Sees teachers smoking in school buildings | 29.7 | 25.8 |
| Regards quitting smoking as difficult (n=10 937) | 37.4 | 21.8 |
| Mean age (years) | 14.8 (SD 1.47) | 15.1 (SD 1.50) |
| Mean experimentation age (years) | | 11.4 (SD 2.96) |

The study employs a duration (or survival) model to estimate the probability of a respondent experimenting with smoking. Survival analysis allows us to analyse the length of time until the



For peer review only - http://bmjopen.bmj.com/site/about/guidelines.xhtml

occurrence of a well-defined end point of interest,[37] which in this case is smoking experimentation. Experimentation is defined in terms of the first time the respondent smoked (at least part of) a cigarette. It is obtained from the question: “Have you ever tried or experimented with cigarette smoking, even one or two puffs?”. The timing of the transition from having never smoked into experimentation depends on the probability of experiencing a transition in period *t,* conditional on not having experienced a transition until period *t*; is also known as the hazard rate or conditional failure rate.[16] The estimation approach requires longitudinal data, making it necessary to transform the cross-sectional GYTS data into pseudo-longitudinal data.[16, 19, 38] The transformation allows for the analysis of time-to-event data. Such data describe the length of time until the occurrence of the event of interest.

As in Asare et al[19], to obtain the longitudinal data required for the duration analysis, we retroactively inferred the year of smoking experimentation using the GYTS question on the age of smoking experimentation: “How old were you when you first tried a cigarette?”. For each age, the individuals were assigned a value of 0 if they did not experiment. The person is assigned a value of 1 in the year in which they tried a cigarette and then drops out of the dataset. The pseudo-longitudinal dataset was constructed on the assumption that a person is at risk of having his/her first cigarette at the age of eight.[19, 38-41] The eldest amongst the respondents was 17 when the survey was conducted in 2017. These respondents were eight years old in 2008. As such, our data sample excluded those who started experimenting with cigarettes before 2008. We also excluded respondents that reported ever-smoking, but who did not provide sufficient data on when they experimented with smoking.



For peer review only - http://bmjopen.bmj.com/site/about/guidelines.xhtml

The pseudo-longitudinal data is then merged with the national-level cigarette price, which allows us to investigate the relationship between price and smoking experimentation. The price data was obtained from The Gambia Bureau of Statistics. The price trend is shown in Figure 1.

*Insert Figure 1 here*

For regression analysis, our baseline model uses a logistic distribution for the hazard function because of its flexibility to allow non-monotone, time-dependent changes of the hazard rate.[16, 42] The regression model estimates the hazard of initiation for individual $i$ in period $t$ ($y_{it}$) as a function of real cigarette prices ($price_t$) and a matrix of explanatory variables ($\boldsymbol{X}_i$), which include socio-demographics (age, gender, parents' and friends' smoking status, income (pocket money) and respondents' perception on quitting smoking). Gender takes the value of 1 for female and 0 for male. Smoking status of the respondent's parents and friends takes the value of 1 if at least one of the parents or friends smokes, and 0 if none of the parents or friends smoke. The respondents' income (pocket money) is derived from the question: "During an average week, how much money do you have that you can spend on yourself, however you want?". The base is 'I usually don't have', and the six other categories are 'Less than GMD25', 'GMD25-GMD50', 'GMD51-GMD100', 'GMD101-GMD150', 'GMD151-GMD200' and 'Above GMD200'. Respondents' perception on quitting smoking is derived from the GYTS question: "Once someone has started smoking tobacco, do you think it would be difficult for them to quit?" The variable is coded as 1 for "Yes" and 0 for "No". The dependent variable, i.e. cigarette smoking experimentation, is extracted from the GYTS data.[34]

The regression model is specified as:

$$y_{it} = Pr\ (initiate|no\ prior\ smoking)$$



For peer review only - http://bmjopen.bmj.com/site/about/guidelines.xhtml

$$= \beta_1 price_t + \beta_2 \boldsymbol{X}_{it} + \varepsilon_{it} \ldots\ldots\ldots (1)$$

We tested the robustness of the results by incorporating a discrete-time split population survival model, which relaxes the assumption that all individuals will eventually smoke. It first estimates each individual's probability of ever experiencing a smoking transition, then weights the hazard function by this probability. The contribution of individual *i* to the log-likelihood function of smoking experimentation is:

$$d_i = ln\{\Pr(ever\ initiate) * f(t \mid t > 0)\} + (1 - d_i) * ln$$
$$\{Pr(ever\ initiate) + Pr(ever\ initiate) * f(t|t = 0)\} \ldots\ldots\ldots (2)$$

where $d_i$ is a binary indicator for experimenting with smoking at some point during the period of observation, *t* is the time of experimentation measured in number of years since age eight, and *f*(*t*) is its probability density function.

As in Vellios and Van Walbeek[38], we used Stata's *spsurv* command for the split population estimation model. The model has been used in numerous studies.[38, 43, 44] The *spsurv* command uses a complementary log-log specification, which reports hazard ratios (not odds ratios) and unlike the logit specification, the response curve is asymmetric.[42] The odds ratios are calculated by taking the antilog of the coefficient. The hazard rate is calculated as (OR / (1 + OR) where OR is the odds ratio. All analyses were done with Stata v.15.

**Patients and public involvement**

Patients and the public were not involved in this study.



For peer review only - http://bmjopen.bmj.com/site/about/guidelines.xhtml

## Results

The regression results are shown in Table 2. The estimated odds and hazard ratios are in exponentiated form and are broadly similar. The hazard ratios are indicated in the table but are not discussed in this section.

**Table 2: Regression results: logistic and split population survival models**

| | Logistic regression model | | Split population model |
|---|---|---|---|
| | Coefficients | Odds ratios | Hazard ratios |
| | (1) | (2) | (3) |
| Price elasticity of experimentation | -0.680** | 0.507** | 0.524*** |
| | (0.302) | (0.153) | (0.131) |
| Parent(s) smoke(s) | 0.371*** | 1.449*** | 1.474*** |
| | (0.082) | (0.120) | (0.093) |
| Friend(s) smoke(s) | 0.482*** | 1.620*** | 1.828*** |
| | (0.073) | (0.119) | (0.102) |
| Difficulty of quitting | 0.055 | 1.056 | 1.114* |
| | (0.069) | (0.073) | (0.062) |
| Teachers smoke in school buildings | 0.176** | 1.192** | 1.205*** |
| | (0.073) | (0.087) | (0.068) |
| Usually do not have pocket money | 0.000 | 1.000 | 1.000 |
| Less than GMD25 | 0.083 | 1.087 | 1.113 |
| | (0.127) | (0.138) | (0.109) |
| GMD25-GMD50 | 0.067 | 1.070 | 1.181* |
| | (0.125) | (0.134) | (0.114) |
| GMD51-GMD100 | 0.334** | 1.397** | 1.400*** |
| | (0.141) | (0.197) | (0.158) |
| GMD101-GMD150 | 0.080 | 1.084 | 1.224 |
| | (0.190) | (0.206) | (0.190) |
| GMD151-GMD200 | 0.246 | 1.279 | 1.396* |
| | (0.227) | (0.290) | (0.261) |
| Above GMD200 | 0.411** | 1.508** | 1.505*** |
| | (0.181) | (0.273) | (0.224) |
| Female | -1.403*** | 0.246*** | 1.159*** |
| | (0.083) | (0.020) | (0.016) |
| Age | 0.165*** | 1.179*** | 1.159*** |
| | (0.020) | (0.024) | (0.016) |
| Constant | -3.426*** | 0.033*** | 0.028*** |
| | (0.892) | (0.029) | (0.021) |



For peer review only - http://bmjopen.bmj.com/site/about/guidelines.xhtml

For peer review only

| Observations | 77,640 | 77,640 | 77,640 |
|---|---|---|---|

Standard errors in parentheses
*** p<0.01, ** p<0.05, * p<0.10

The results show that a 1% increase in the price of cigarettes reduces the probability to experiment with smoking among children by 0.7% (95% CI: -1.1 to -0.4) (as indicated in column 1).

Children with at least one smoking parent are more likely to have ever smoked a cigarette than those whose parents do not smoke (OR = 1.4, 95% CI: 1.2 to 1.7). Also, children with friends who smoke are more likely to have ever experimented with smoking a cigarette than those whose friends do not smoke (OR = 1.6, 95% CI: 0.4 to 1.9).

Children who see their teachers smoking inside school buildings are more likely to have ever experimented with smoking a cigarette than those who do not see teachers smoking inside school buildings (OR = 1.2, 95% CI: 1.0 to 1.4). Income (i.e., having access to pocket money) did not have a clear effect, although there is some suggestion that children who receive pocket money are more likely to experiment with smoking than children who do not receive pocket money.[45]

Female youths are less likely to have ever smoked a cigarette than male youths (OR = 0.2, 95% CI: 0.2 to 0.3). The results also show that a one-year increase in age increases the probability to experiment with smoking (OR = 1.2, 95% CI:1.1 to 1.2). Figure 2 show the smoking initiation hazard rates by age and gender. The hazard rates are derived from the regression analyses.

*Insert Figure 2 here*

Figure 2 shows a positive correlation between age and the probability to experiment with smoking cigarettes among the youth in The Gambia. The probability to experiment with smoking is higher among boys than among girls.



For peer review only - http://bmjopen.bmj.com/site/about/guidelines.xhtml

## Discussion

The price elasticity of demand for smoking experimentation is estimated to be -0.7 among children in The Gambia. This implies that a 10% rise in the price of cigarettes is associated with a 7% reduction in the probability to experiment with smoking among the Gambian children. Increasing excise taxes could be a highly effective tobacco-control tool in The Gambia. The excise tax typically increases the retail price of cigarettes, thereby reducing the propensity to experiment with smoking.[1, 16] Considering that the current tax burden of 46.3% is well below the 75% target recommended by the World Health Organisation,[46] there is room for the government to further raise the excise tax on tobacco. Raising tobacco tax is a 'win-win' policy, as it generates extra revenue for the government while discouraging tobacco consumption through higher prices, thereby improving public health.[29]

The study established that children with smoking friends were more likely to have ever smoked a cigarette than those whose friends do not smoke. Similarly, children from households with at least one parent who currently smokes were more likely to have ever smoked a cigarette. This could be because, as children are more exposed to smoking (especially by parents) they would regard smoking as normal. Also, if the parents smoke, access to cigarettes is easier, than if the parents do not smoke. The results from this study are in line with the findings of Jallow et al[47] who found that children in smoking families were more likely to smoke than those in non-smoking families. We also found that children are more likely to experiment with cigarette smoking if they see their teachers smoking in school buildings. If smoke-free regulations are effectively implemented, it would presumably reduce smoking in school buildings, and thus reducing youth smoking. Nevertheless, our study found that peer influence was more important in experimenting with smoking than both parental and teachers' influence. A systematic review of studies conducted



For peer review only - http://bmjopen.bmj.com/site/about/guidelines.xhtml

among high school and university students in Ethiopia and other studies in the US found that peer pressure had a significant influence on young peoples' initiation and smoking of cigarettes.[33, 48] Increases in the price will have a direct and indirect influence on smoking. The direct influence will be through the price elasticity of demand. The indirect influence will be through the peers. If the price increases, the peers will smoke less (or not at all), thus decreasing the peer pressure.

The Gambian youths start smoking as early as at age eight, a result similar to a previously published study.[26] The likelihood of smoking increases exponentially as age increases. As in most other countries, the probability of smoking is higher among boys than among girls. This is mirrored by the smoking prevalence in the general population, where smoking prevalence among women is very low (about 1%) compared to men (32.1%).[26] However, the smoking rate among girls is substantially higher than among adult women, suggesting that female smoking prevalence may increase sharply in future, as girls step into adulthood. This would be detrimental to public health. The government should implement policies, including an increase in the excise tax, that will prevent an increase in smoking experimentation.

### Limitation of the study

Although this study provides useful information for devising suitable tobacco-tax policy measures, there are some limitations to consider. For instance, the study relied on individuals' own memory regarding past smoking behaviour. As such, the dependent variable could be subject to measurement error with an unknown bias. However, this challenge is more prevalent among adult respondents.[16, 19] Considering that our sample was composed of children, with relatively short smoking histories, we believe that our findings are robust. In addition, the study relied on a pseudo-panel where several individual demographic and socio-economic characteristics were fixed at the time of the survey. To the extent that some of these socio-economic variables have changed in the



For peer review only - http://bmjopen.bmj.com/site/about/guidelines.xhtml

years prior to the survey, this could have biased the results. Also, considering that the 2017 GYTS is a cross-sectional survey among students in junior secondary schools, the data may not be a good representation of Gambian youth, e.g. those in senior secondary schools (Grades 10-12) and school dropouts. We do not have information on what happens beyond this age group, because the data are not collected. In addition, some important information such as the respondents' region, race, and religion were not collected. Had these variables been available, they could have been used in the regression analysis.

## Conclusion

We found the price elasticity of demand for smoking experimentation to be -0.7 among children who have smoked at least once or twice in their lives. Our results indicate that an increase in tobacco excise taxes can play an effective role in discouraging children from experimenting with smoking. Considering the current relatively low excise tax burden, the government should consider increasing the excise tax burden on tobacco products in line with the recommendations of the World Health Organisation.[32]

**Acknowledgements:** Special thanks to Nicole Vellios who reviewed earlier drafts.

**Contributors:** CD and MKB conceptualised the study and conducted the data analysis. CD, MKB and CB wrote the first draft of the manuscript. CvW, ZG and UD'A contributed to the drafting and revision of the manuscript.

**Funding:** This study was primarily supported by the UK Research and Innovation (UKRI) with funding from the Global Challenges Research Fund (MR/P027946/2), as part of the Tobacco Control Capacity Programme (TCCP). The TCCP is a programme of capacity development and



For peer review only - http://bmjopen.bmj.com/site/about/guidelines.xhtml

research co-ordinated by the University of Edinburgh, Scotland and involves 15 partner institutions from Africa, South Asia and the United Kingdom. The study was also supported by the South African Medical Research Council (grant no: 23108).

**Competing interests:** None.

**Patient consent for publication:** Not required.

**Ethics approval:** N/A. The data is publicly available.

**Data sharing statement:** Data are publicly available on: https://nccd.cdc.gov/GTSSDataSurveyResources/Ancillary/DataReports.aspx?CAID=2.

## References

[1] National Cancer Institute and World Health Organization. The Economics of Tobacco and Tobacco Control: National Cancer Institute Tobacco Control Monograph 21 NIH Publication No 16-CA-8029A: Bethesda, MD: U.S. Department of Health and Human Services, National Institutes of Health, National Cancer Institute; and Geneva, CH: World Health Organization; 2016. Available from: https://cancercontrol.cancer.gov/sites/default/files/2020-06/m21_complete.pdf.

[2] World Health Organization. Global Health Observatory data: prevalence of tobacco smoking. Web address: http://wwwwhoint/gho/tobacco/use/en/, Accessed date. 2020;28.

[3] Bilano V, Gilmour S, Moffiet T, d'Espaignet ET, Stevens GA, Commar A, et al. Global trends and projections for tobacco use, 1990–2025: an analysis of smoking indicators from the WHO Comprehensive Information Systems for Tobacco Control. The Lancet. 2015;385(9972):966-76.



For peer review only - http://bmjopen.bmj.com/site/about/guidelines.xhtml

[4] World health Organization. Tobacco Factsheet 2020 [Available from: https://www.who.int/en/news-room/fact-sheets/detail/tobacco [Accessed 02 March 2021].

[5] Forouzanfar MH, Afshin A, Alexander LT, Anderson HR, Bhutta ZA, Biryukov S, et al. Global, regional, and national comparative risk assessment of 79 behavioural, environmental and occupational, and metabolic risks or clusters of risks, 1990–2015: a systematic analysis for the Global Burden of Disease Study 2015. The lancet. 2016;388(10053):1659-724.

[6] Blecher EH, Ross H. Tobacco use in Africa: tobacco control through prevention. American Cancer Society. 2015.

[7] Chandora R, Song Y, Chaussard M, Palipudi KM, Lee KA, Ramanandraibe N, et al. Youth access to cigarettes in six sub-Saharan African countries. Preventive Medicine. 2016;91:S23-S7.

[8] Organization WH. WHO report on the global tobacco epidemic, 2013: enforcing bans on tobacco advertising, promotion and sponsorship: World Health Organization; 2013.

[9] World Health Organization. WHO global report on trends in prevalence of tobacco smoking 2000-2025: World Health Organization; 2018.

[10] Sargent JD, Gabrielli J, Budney A, Soneji S, Wills TA. Adolescent smoking experimentation as a predictor of daily cigarette smoking. Drug and Alcohol Dependence. 2017;175:55-9.

[11] Friedman AS, Buckell J, Sindelar JL. Patterns of youth cigarette experimentation and onset of habitual smoking. American journal of preventive medicine. 2019;56(6):803-10.

[12] Ho L-M, Schafferer C, Lee J-M, Yeh C-Y, Hsieh C-J. The effect of cigarette price increases on cigarette consumption, tax revenue, and smoking-related death in Africa from 1999 to 2013. International journal of public health. 2017;62(8):899-909.

For peer review only



For peer review only - http://bmjopen.bmj.com/site/about/guidelines.xhtml

[13] Boachie MK, Immurana M, Agyemang JK, Ross H. Cigarette prices and smoking experimentation in Sierra Leone: an exploratory study. Tobacco Use Insights. 2022;15:1179173X221078189.

[14] Mukong AK, Tingum EN. The demand for cigarettes: New evidence from South Africa. Development Southern Africa. 2020;37(1):40-54.

[15] Kidane A, Mduma J, Naho A, Ngeh ET, Hu T-w. The demand for cigarettes in tanzania and implications for tobacco taxation policy. Advances in economics and business. 2015;3(10):428.

[16] Dauchy E, Ross H. The effect of price and tax policies on the decision to smoke among men in Kenya. Addiction. 2019;114(7):1249-63.

[17] Stoklosa M, Goma F, Nargis N, Drope J, Chelwa G, Chisha Z, et al. Price, tax and tobacco product substitution in Zambia: findings from the ITC Zambia Surveys. Tobacco Control. 2019;28(Suppl 1):s45-s52.

[18] Chelwa G, Van Walbeek C. Does cigarette demand respond to price increases in Uganda? Price elasticity estimates using the Uganda National Panel Survey and Deaton's method. BMJ open. 2019;9(3):e026150.

[19] Asare S, Stoklosa M, Drope J, Larsen A. Effects of Prices on Youth Cigarette Smoking and Tobacco Use Initiation in Ghana and Nigeria. International journal of environmental research and public health. 2019;16(17):3114.

[20] United Nations Development Programme. Human Development Insight 2022 [Available from: https://hdr.undp.org/data-center/country-insights#/ranks.



For peer review only - http://bmjopen.bmj.com/site/about/guidelines.xhtml

[21] World Bank. GDP per capita (current US$) - Lower middle income, Gambia, The. 2022 [Available from: https://data.worldbank.org/indicator/NY.GDP.PCAP.CD?locations=XN-GM.

[22] United Nations. United Nations Treat Collections. 2022 [Available from: https://treaties.un.org/pages/ViewDetails.aspx?src=TREATY&mtdsg_no=IX-4&chapter=9&clang=_en.

[23] World Health Organization. Legislation by country: Gambia 2022 [Available from: https://www.tobaccocontrollaws.org/legislation/country/gambia/summary#:~:text=The%20law%20prohibits%20the%20sale,worship%2C%20and%20other%20specified%20locations.

[24] Cham B, Mdege ND, Bauld L, Britton J, D'alessandro U. Exposure to Second-Hand Smoke in Public Places and Barriers to the Implementation of Smoke-Free Regulations in The Gambia: A Population-Based Survey. International journal of environmental research and public health. 2021;18(12):6263.

[25] Cham B, Scholes S, Ng Fat L, Badjie O, Mindell JS. Burden of hypertension in The Gambia: evidence from a national World Health Organization (WHO) STEP survey. International journal of epidemiology. 2018;47(3):860-71.

[26] Cham B, Scholes S, Groce NE, Mindell JS. Prevalence and predictors of smoking among Gambian men: a cross-sectional national WHO STEP survey. International journal of environmental research and public health. 2019;16(23):4719.

[27] Jallow IK, Britton J, Langley T. Prevalence and determinants of tobacco use among young people in The Gambia. BMJ global health. 2017;2(4).

[28] Manneh E, Gambia. A Global Youth Tobacco Survey (GYTS) Country Report 2008. Government of The Gambia: Banjul, The Gambia. 2008.



For peer review only - http://bmjopen.bmj.com/site/about/guidelines.xhtml

[29] Nargis N, Manneh Y, Krubally B, Jobe B, Ouma AEO, Tcha-Kondor N, et al. How effective has tobacco tax increase been in the Gambia? A case study of tobacco control. BMJ open. 2016;6(8):e010413.

[30] The Gambia Bureau of Statistics. 2020.

[31] World Health Organization. WHO report on the global tobacco epidemic 2019: Offer help to quit tobacco use. Geneva: World Health Organization; 2019.

[32] World Health Organization. Taxation 2020 [Available from: https://www.euro.who.int/en/health-topics/disease-prevention/tobacco/publications/key-policy-documents/who-framework-convention-on-tobacco-control-who-fctc/key-areas-of-tobacco-control-policy/taxation Access: 20 July 2020.

[33] IARC. IARC handbooks of cancer prevention, tobacco control, vol. 14: effectiveness of tax and price policies for tobacco control. Lyon, France. 2011.

[dataset] [34] Centers for Disease Control and Prevention. Global Tobacco Surveillance System Data. 2021 [Available from: https://nccd.cdc.gov/GTSSDataSurveyResources/Ancillary/DataReports.aspx?CAID=2 [Accessed: 23 January 2021].

[35] Cadmus EO, Ayo-Yusuf OA. The effect of smokeless tobacco use and exposure to cigarette promotions on smoking intention among youths in Ghana. Cogent Medicine. 2018;5(1):1531459.

[36] Boachie MK, Immurana M, Tingum EN, Mdege ND, Ross H. Effect of relative income price on smoking initiation among adolescents in Ghana: evidence from pseudo-longitudinal data. BMJ open. 2022;12(3):e054367.



For peer review only - http://bmjopen.bmj.com/site/about/guidelines.xhtml

[37] Schober P, Vetter TR. Survival analysis and interpretation of time-to-event data: the tortoise and the hare. Anesthesia and analgesia. 2018;127(3):792.

[38] Vellios N, van Walbeek C. Determinants of regular smoking onset in South Africa using duration analysis. BMJ open. 2016;6(7).

[39] Guindon GE, Paraje GR, Chávez R. Prices, inflation, and smoking onset: the case of Argentina. Economic Inquiry. 2018;56(1):424-45.

[40] Marti J. The impact of tobacco control expenditures on smoking initiation and cessation. Health economics. 2014;23(12):1397-410.

[41] Madden D. Tobacco taxes and starting and quitting smoking: does the effect differ by education? Applied Economics. 2007;39(5):613-27.

[42] Lambert PC. Modeling of the cure fraction in survival studies. The Stata Journal. 2007;7(3):351-75.

[43] Lillard DR, Molloy E, Sfekas A. Smoking initiation and the iron law of demand. Journal of health economics. 2013;32(1):114-27.

[44] Kidd MP, Hopkins S. The hazards of starting and quitting smoking: some Australian evidence. Economic Record. 2004;80(249):177-92.

[45] Cui Y, Forget EL, Zhu Y, Torabi M, Oguzoglu U. The effects of cigarette price and the amount of pocket money on youth smoking initiation and intensity in Canada. Canadian Journal of Public Health. 2019;110(1):93-102.

[46] World Health Organization. Taxation 2021 [Available from: https://www.euro.who.int/en/health-topics/disease-prevention/tobacco/publications/key-policy-documents/who-framework-convention-on-tobacco-control-who-fctc/key-areas-of-tobacco-control-policy/taxation.



For peer review only - http://bmjopen.bmj.com/site/about/guidelines.xhtml

[47] Jallow IK, Britton J, Langley T. Prevalence and determinants of susceptibility to tobacco smoking among students in the Gambia. Nicotine and Tobacco Research. 2019;21(8):1113-21.

[48] Leshargie CT, Alebel A, Kibret GD, Birhanu MY, Mulugeta H, Malloy P, et al. The impact of peer pressure on cigarette smoking among high school and university students in Ethiopia: A systemic review and meta-analysis. PloS one. 2019;14(10):e0222572.

For peer review only

**Figure 1: Average real price, 2008 – 2017**

**Figure 2: Smoking hazard rates for smoking experimentation**



For peer review only - http://bmjopen.bmj.com/site/about/guidelines.xhtml

**Figure 1: Average real price, 2008 – 2017**

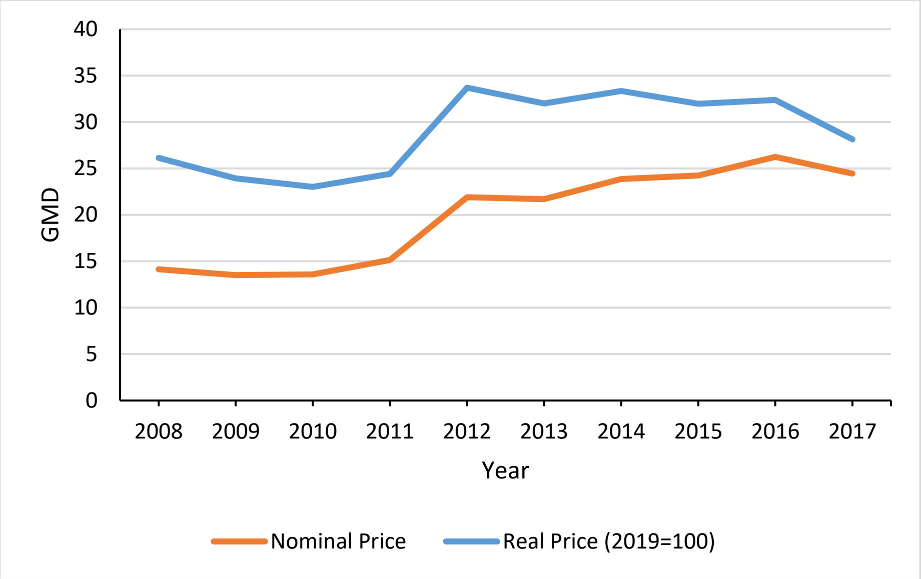


**Source:** The Gambia Bureau of Statistics

For peer review only

**Figure 2: Smoking hazard rates for smoking experimentation**

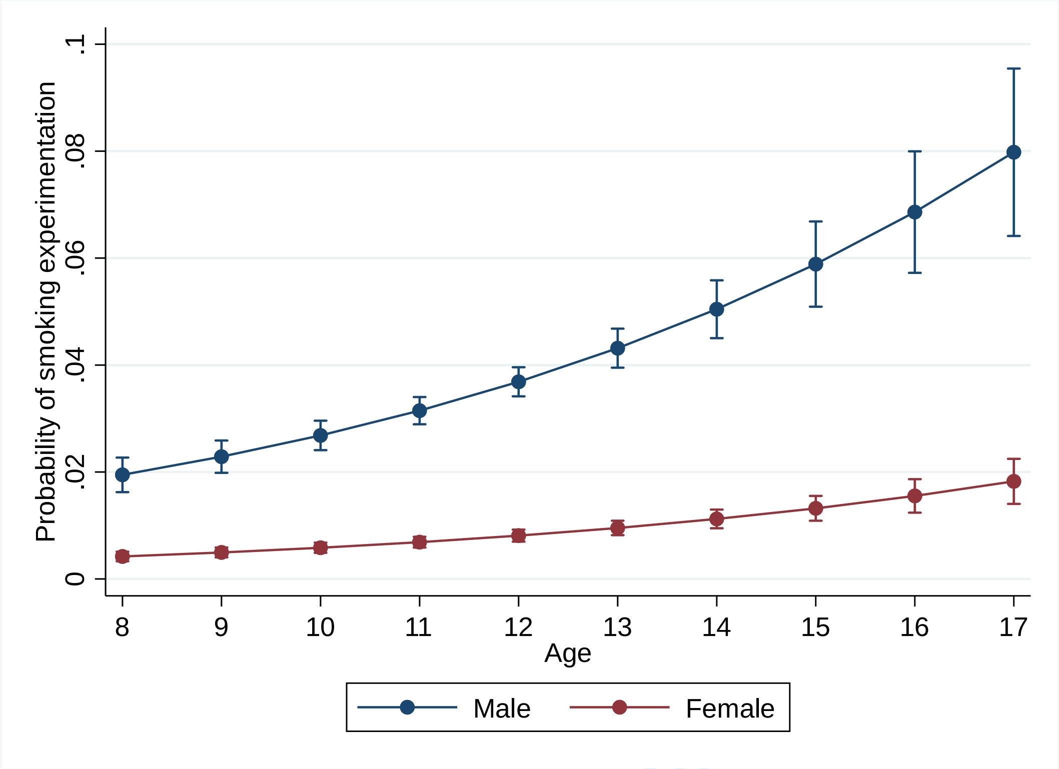

STROBE Statement—Checklist of items that should be included in reports of ***cross-sectional studies***

| | Item No | Recommendation | Page No |
|---|---|---|---|
| **Title and abstract** | 1 | (*a*) Indicate the study's design with a commonly used term in the title or the abstract | 2 |
| | | (*b*) Provide in the abstract an informative and balanced summary of what was done and what was found | 3 |
| **Introduction** | | | |
| Background/rationale | 2 | Explain the scientific background and rationale for the investigation being reported | 4-6 |
| Objectives | 3 | State specific objectives, including any prespecified hypotheses | 6 |
| **Methods** | | | |
| Study design | 4 | Present key elements of study design early in the paper | 7 |
| Setting | 5 | Describe the setting, locations, and relevant dates, including periods of recruitment, exposure, follow-up, and data collection | 7-8 |
| Participants | 6 | (*a*) Give the eligibility criteria, and the sources and methods of selection of participants | 8 |
| Variables | 7 | Clearly define all outcomes, exposures, predictors, potential confounders, and effect modifiers. Give diagnostic criteria, if applicable | 8-9 |
| Data sources/ measurement | 8* | For each variable of interest, give sources of data and details of methods of assessment (measurement). Describe comparability of assessment methods if there is more than one group | 8-9 |
| Bias | 9 | Describe any efforts to address potential sources of bias | 10 |
| Study size | 10 | Explain how the study size was arrived at | 8 |
| Quantitative variables | 11 | Explain how quantitative variables were handled in the analyses. If applicable, describe which groupings were chosen and why | 9 |
| Statistical methods | 12 | (*a*) Describe all statistical methods, including those used to control for confounding | 9-10 |
| | | (*b*) Describe any methods used to examine subgroups and interactions | 9-10 |
| | | (*c*) Explain how missing data were addressed | 8 |
| | | (*d*) If applicable, describe analytical methods taking account of sampling strategy | 7-8 |
| | | (*e*) Describe any sensitivity analyses | 9 |
| **Results** | | | |
| Participants | 13* | (a) Report numbers of individuals at each stage of study—eg numbers potentially eligible, examined for eligibility, confirmed eligible, included in the study, completing follow-up, and analysed | 8 |
| | | (b) Give reasons for non-participation at each stage | |
| | | (c) Consider use of a flow diagram | |
| Descriptive data | 14* | (a) Give characteristics of study participants (eg demographic, clinical, social) and information on exposures and potential confounders | 7 |
| | | (b) Indicate number of participants with missing data for each variable of interest | |
| Outcome data | 15* | Report numbers of outcome events or summary measures | |
| Main results | 16 | (*a*) Give unadjusted estimates and, if applicable, confounder-adjusted estimates and their precision (eg, 95% confidence interval). Make clear which confounders were adjusted for and why they were included | 11-12 |



For peer review only - http://bmjopen.bmj.com/site/about/guidelines.xhtml

| | | | |
|---|---|---|---|
| | | (*b*) Report category boundaries when continuous variables were categorized | |
| | | (*c*) If relevant, consider translating estimates of relative risk into absolute risk for a meaningful time period | |
| Other analyses | 17 | Report other analyses done—eg analyses of subgroups and interactions, and sensitivity analyses | |
| **Discussion** | | | |
| Key results | 18 | Summarise key results with reference to study objectives | 10-12 |
| Limitations | 19 | Discuss limitations of the study, taking into account sources of potential bias or imprecision. Discuss both direction and magnitude of any potential bias | 14 |
| Interpretation | 20 | Give a cautious overall interpretation of results considering objectives, limitations, multiplicity of analyses, results from similar studies, and other relevant evidence | 12-13 |
| Generalisability | 21 | Discuss the generalisability (external validity) of the study results | 14 |
| **Other information** | | | |
| Funding | 22 | Give the source of funding and the role of the funders for the present study and, if applicable, for the original study on which the present article is based | 15 |

*Give information separately for exposed and unexposed groups.

**Note:** An Explanation and Elaboration article discusses each checklist item and gives methodological background and published examples of transparent reporting. The STROBE checklist is best used in conjunction with this article (freely available on the Web sites of PLoS Medicine at http://www.plosmedicine.org/, Annals of Internal Medicine at http://www.annals.org/, and Epidemiology at http://www.epidem.com/). Information on the STROBE Initiative is available at www.strobe-statement.org.

For peer review only


For peer review only - http://bmjopen.bmj.com/site/about/guidelines.xhtml

BMJ Open

# The effect of price on the decision to experiment with cigarette smoking among Gambian children: A survival analysis using the Gambia 2017 Global Youth Tobacco Survey data

| | |
|---|---|
| Journal: | *BMJ Open* |
| Manuscript ID | bmjopen-2022-061045.R2 |
| Article Type: | Original research |
| Date Submitted by the Author: | 10-Oct-2022 |
| Complete List of Authors: | Dare, Chengetai; University of Cape Town, Research Unit on the Economics of Excisable Products (REEP), School of Economics<br>Cham, Bai; Medical Research Council Unit The Gambia at the London School of Hygiene and Tropical Medicine<br>Boachie, Micheal Kofi; University of the Witwatersrand Faculty of Health Sciences, SAMRC/Wits Centre for Health Economics and Decision Science -PRICELESS SA; University of Cape Town, Research Unit on the Economics of Excisable Products<br>Gitonga, Zachary; University of Cape Town, Research Unit on the Economics of Excisable Products (REEP), School of Economics<br>D'Alessandro, Umberto; Medical Research Council Unit The Gambia at the London School of Hygiene and Tropical Medicine<br>Walbeek, Corne; University of Cape Town, Research Unit on the Economics of Excisable Products, School of Economics |
| <b>Primary Subject Heading</b>: | Smoking and tobacco |
| Secondary Subject Heading: | Public health, Health policy, Health economics |
| Keywords: | HEALTH ECONOMICS, Health policy < HEALTH SERVICES ADMINISTRATION & MANAGEMENT, Health economics < HEALTH SERVICES ADMINISTRATION & MANAGEMENT, PUBLIC HEALTH |
| | |

SCHOLARONE™
Manuscripts

*I, the Submitting Author has the right to grant and does grant on behalf of all authors of the Work (as defined in the below author licence), an exclusive licence and/or a non-exclusive licence for contributions from authors who are: i) UK Crown employees; ii) where BMJ has agreed a CC-BY licence shall apply, and/or iii) in accordance with the terms applicable for US Federal Government officers or employees acting as part of their official duties; on a worldwide, perpetual, irrevocable, royalty-free basis to BMJ Publishing Group Ltd ("BMJ") its licensees and where the relevant Journal is co-owned by BMJ to the co-owners of the Journal, to publish the Work in this journal and any other BMJ products and to exploit all rights, as set out in our licence.*

*The Submitting Author accepts and understands that any supply made under these terms is made by BMJ to the Submitting Author unless you are acting as an employee on behalf of your employer or a postgraduate student of an affiliated institution which is paying any applicable article publishing charge ("APC") for Open Access articles. Where the Submitting Author wishes to make the Work available on an Open Access basis (and intends to pay the relevant APC), the terms of reuse of such Open Access shall be governed by a Creative Commons licence – details of these licences and which Creative Commons licence will apply to this Work are set out in our licence referred to above.*

*Other than as permitted in any relevant BMJ Author's Self Archiving Policies, I confirm this Work has not been accepted for publication elsewhere, is not being considered for publication elsewhere and does not duplicate material already published. I confirm all authors consent to publication of this Work and authorise the granting of this licence.*

For peer review only

# The effect of price on the decision to experiment with cigarette smoking among Gambian children: A survival analysis using the Gambia 2017 Global Youth Tobacco Survey data

Chengetai Dare[1 *], Cham Bai[2], Micheal K. Boachie[1,3], Zachary Gitonga[1], Umberto D'Alessandro[2], Corné van Walbeek[1]

[1] Research Unit on the Economics of Excisable Products (REEP), School of Economics, University of Cape Town, Private Bag X3, Rondebosch 7700, South Africa

[*] Email: cdarejam@yahoo.com; Fax: +27 (0)21 650 4369; Mobile: +27 (0)61 880 8197

[2] Disease Control and Elimination Theme, Medical Research Council Unit The Gambia at the London School of Hygiene and Tropical Medicine, Atlantic Road, Fajara, P.O. Box 273, Banjul, The Gambia

[3] SAMRC/Wits Centre for Health Economics and Decision Science – PRICELESS SA, Wits School of Public Health, Faculty of Health Sciences, University of the Witwatersrand, Parktown, Johannesburg, 2193, South Africa.

**Keywords:** smoking experimentation, price elasticity, children, demand for cigarettes, excise tax, The Gambia

**Word count:** 3215

For peer review only

# The effect of price on the decision to experiment with cigarette smoking among Gambian children: A survival analysis using the Gambia 2017 Global Youth Tobacco Survey data


## Abstract

**Objectives:** To investigate the relationship between cigarette prices and smoking experimentation among children in The Gambia, and thereby expanding the evidence base of the likely impact of excise taxes on cigarette demand in low-income and middle-income countries.

**Design:** A survival analysis using the Gambia 2017 Global Youth Tobacco Survey data.

**Setting:** The Gambia.

**Participants:** The survey sample was 12,585 youths, aged 12 – 17 years but our analysis was restricted to 11,030 respondents with information on smoking status.

**Primary and secondary outcome measures:** Our outcome variable was smoking experimentation defined as the first time the respondent smoked (at least part of) a cigarette.

**Results:** A 1% increase in the price of cigarettes reduces the probability to experiment with smoking by 0.7%. We also found that children are more likely to experiment with smoking if they have at least one smoking parent, friends who smoke, and see teachers who smoke. The probability to experiment with cigarette smoking increases with age and is higher among boys than girls.

**Conclusion:** There is strong evidence that increasing excise taxes can play an effective role in discouraging children from experimenting with cigarette smoking. Considering the relatively low excise tax burden in the Gambia, the government should consider substantially increasing the excise tax burden.


**Strengths and limitations of this study**



For peer review only - http://bmjopen.bmj.com/site/about/guidelines.xhtml

- Survival analysis using pseudo-longitudinal data (obtained from cross sectional data) is a good strategy in estimating longitudinal outcomes in settings where longitudinal studies are costly and/or difficult to conduct.
- There are very few tobacco demand studies in Sub-Saharan Africa that employed survival analysis, and this is the first in the Gambia.
- The Global Youth Tobacco Survey is cross-sectional and focuses on students in junior secondary schools, the data may not be a good representation of Gambian youth, including those in senior secondary schools, children out of school and school dropouts.
- The study relied on individuals' own memory regarding past smoking behaviour and hence the dependent variable could be subject to measurement error (specifically, recall bias).

For peer review only

## Introduction

Smoking is the leading cause of preventable death.[1] There are over one billion smokers globally and around 80% of them live in low- and middle-income countries (LMICs).[1, 2] Africa's share of the global number of smokers is rapidly increasing.[3] Over 8 million people die every year from tobacco use and/or exposure to tobacco smoke,[2, 4] and 80% of all tobacco-related deaths by 2030 are predicted to occur in LMICs.[5, 6] Tobacco companies are shifting their target to LMICs to build a broader consumer base, especially among young people.[7, 8] In the WHO African region, the total number of smokers is projected to increase by 27.3% from 66 million in 2015 to 84 million by 2025;[9] this is the second-largest percentage increase after the Eastern Mediterranean region.

Research has shown that experimentation with cigarettes among young people is associated with future daily smoking.[10, 11] In a study among 6522 US adolescents aged 10 to 14 years, experimentation with cigarette smoking during ages 10-14 years predicted daily smoking two years later, independent of other cigarette smoking risk factors.[10] Evidence has also shown that higher prices is a deterrent of cigarette use among young people, especially in Africa. In several African countries, higher cigarette prices have contributed to a reduction of both tobacco use prevalence and intensity.[12] This has been confirmed in country-specific studies in Sierra Leone,[13] South Africa,[14] Tanzania,[15] Kenya,[16] Zambia,[17] and Uganda.[18] Similarly, studies have found that, in Ghana and Nigeria, increased cigarette prices decreased both smoking intensity and smoking initiation.[19]

The Gambia is the smallest country on mainland Africa with a population of about 2 million. It is a low-income country and is ranked 172 out of 189 countries in the 2020 United Nations Human Development Index,[20] with per capita GDP of $773.[21] The country ratified the WHO



For peer review only - http://bmjopen.bmj.com/site/about/guidelines.xhtml

Framework Convention on Tobacco Control (WHO FCTC) in September 2007.[22] There are several regulations and policies on tobacco control in The Gambia, including the Prohibition of Smoking in Public Places Act 1998, the Tax Policy Reform Act 2013, the Tobacco Control Act 2016 and the Tobacco Control Regulations 2019.[23, 24] These legal instruments seek to protect children from exposure to tobacco products. However, despite the numerous achievements in tobacco control as highlighted above, there are still gaps, especially on the implementation of smoke-free regulation in The Gambia.[24] There has also been some regression, specifically with the Tax Policy Reform Act, which has been stalled since 2017, following a change in government.

The prevalence of smoking is 15.9% among Gambian adults (25-64 years old),[25] 1% among women, and 32.1% among men.[26] A 2016 survey conducted among secondary school students (12-20 years) indicated that 7.9% of boys and 1.5% of girls had smoked at least once in the past 30 days.[27] An earlier 2008 GYTS survey indicated that 10.8% of 13-15-year-old students (12.7% among boys and 8.6% among girls) had smoked at least once in the past 30 days.[28]

Like other governments across the world, The Gambian government employs tax and price measures to reduce the affordability of tobacco. For instance, in 2013 a specific excise tax was introduced on all imported tobacco products. This contributed to an increase in cigarette prices and tax revenue and to a reduction in tobacco imports.[29] However, cigarette prices remain relatively low. Using 2019 exchange rates, data obtained from The Gambia Bureau of Statistics[30] show that the average retail price for a packet of 20 sticks (of the most popular brands) ranged between GMD26.15 (US$0.51) and GMD31.70 between 2008 and 2019, which is substantially lower than the sub-Saharan African regional average of US$1.80, and the global average price of US$3.82.[31] The tax burden (share of taxes in the retail price) was 46.3% in 2018,[31] which is below the 75% target recommended by the World Health Organisation.[32]



For peer review only - http://bmjopen.bmj.com/site/about/guidelines.xhtml

For peer review only

An increase in the excise tax is the single most important intervention to reduce smoking prevalence and intensity.[32, 33] Youth are even more price-sensitive than adults, making tobacco taxation particularly effective; this is even more so in countries with a young population.[19] Although global knowledge about the effect of prices (or taxes) as a tobacco control measure is well established, according to our knowledge, there is no evidence on the impact of prices on tobacco use experimentation among the children in the Gambia. In this paper we fill this knowledge gap by employing survival analysis to examine the effect of prices on the decision to experiment with cigarette smoking among children in The Gambia.

## Data and methodology

We used the 2017 Global Youth Tobacco Survey (GYTS)[34] to obtain individual and household information on smoking behaviour and other background characteristics. The GYTS is a nationally representative school-based survey designed by the Centre for Disease Control and Prevention (CDC) as a global standard tool for monitoring tobacco use among youth and to guide the implementation and evaluation of tobacco prevention and control programmes.[34] The survey considers a cross-section of students in junior secondary schools (Grades 7-9). It does not follow individuals over time, but provides a snapshot on their smoking patterns. The sample is drawn using a two-stage cluster-sampling design.[35, 36] Schools are selected with probability proportional to school enrolment size during the first stage, and then classes within participating schools are selected as a systematic equal probability sample with a random start during the second stage.[36] All students in the selected classes are eligible to participate in the survey.[34, 36]

The 2017 GYTS covers a sample of 12,585 youths, aged 12 – 17 years. However, 1,555 respondents did not provide sufficient information about their smoking status, thereby reducing



For peer review only - http://bmjopen.bmj.com/site/about/guidelines.xhtml

our sample size to 11,030. Smoking experimenters or ever-smokers (defined as having smoked at least once or twice over their lifetimes) was reported by 2,218 (20.1%) respondents (Table 1).

**Table 1: Demographic characteristics and smoking habits (%)**

| | N (and proportion of total sample) | Ever-smokers in the category |
|---|---|---|
| Students sampled (N=11 030) | n = 11 030 | n = 2218 (20.1%) |
| Gender (n=10 886) | | |
| Male | 44.3 | 32.9 |
| Female | 55.7 | 9.6 |
| Age (n=10 483) | | |
| 12 | 6.0 | 16.4 |
| 13 | 14.9 | 15.0 |
| 14 | 23.6 | 16.7 |
| 15 | 21.7 | 18.3 |
| 16 | 17.1 | 22.4 |
| 17 | 16.7 | 28.1 |
| Parent(s) smoke(s) (n= 10 945) | 18.4 | 29.7 |
| Friend(s) smoke(s) (n=10 916) | 23.0 | 38.0 |
| Amount of pocket money per week (n=10 918): | | |
| Usually do not have | 12.1 | 17.4 |
| Less than GMD25 | 35.7 | 19.0 |
| GMD25-GMD50 | 32.5 | 19.8 |
| GMD51-GMD100 | 10.5 | 23.1 |
| GMD101-GMD150 | 3.9 | 23.0 |
| GMD151-GMD200 | 1.9 | 25.0 |
| Above GMD200 | 3.4 | 27.8 |
| Sees teachers smoking in school buildings | 29.7 | 25.8 |
| Regards quitting smoking as difficult (n=10 937) | 37.4 | 21.8 |
| Mean age (years) | 14.8 (SD 1.47) | 15.1 (SD 1.50) |
| Mean experimentation age (years) | | 11.4 (SD 2.96) |

The study employs a duration (or survival) model to estimate the probability of a respondent experimenting with smoking. Survival analysis allows us to analyse the length of time until the



For peer review only - http://bmjopen.bmj.com/site/about/guidelines.xhtml

occurrence of a well-defined end point of interest,[37] which in this case is smoking experimentation. Experimentation is defined in terms of the first time the respondent smoked (at least part of) a cigarette. It is obtained from the question: "Have you ever tried or experimented with cigarette smoking, even one or two puffs?". The timing of the transition from having never smoked into experimentation depends on the probability of experiencing a transition in period *t,* conditional on not having experienced a transition until period *t*; is also known as the hazard rate or conditional failure rate.[16] The estimation approach requires longitudinal data, making it necessary to transform the cross-sectional GYTS data into pseudo-longitudinal data.[16, 19, 38] The transformation allows for the analysis of time-to-event data. Such data describe the length of time until the occurrence of the event of interest.

As in Asare et al[19], to obtain the longitudinal data required for the duration analysis, we retroactively inferred the year of smoking experimentation using the GYTS question on the age of smoking experimentation: "How old were you when you first tried a cigarette?". For each age, the individuals were assigned a value of 0 if they did not experiment. The person is assigned a value of 1 in the year in which they tried a cigarette and then drops out of the dataset. The pseudo-longitudinal dataset was constructed on the assumption that a person is at risk of having his/her first cigarette at the age of eight.[19, 38-41] The eldest amongst the respondents was 17 when the survey was conducted in 2017. These respondents were eight years old in 2008. As such, our data sample excluded those who started experimenting with cigarettes before 2008. We also excluded respondents that reported ever-smoking, but who did not provide sufficient data on when they experimented with smoking.



For peer review only - http://bmjopen.bmj.com/site/about/guidelines.xhtml

The pseudo-longitudinal data is then merged with the national-level cigarette price, which allows us to investigate the relationship between price and smoking experimentation. The price data was obtained from The Gambia Bureau of Statistics. The price trend is shown in Figure 1.

*Insert Figure 1 here*

For regression analysis, our baseline model uses a logistic distribution for the hazard function because of its flexibility to allow non-monotone, time-dependent changes of the hazard rate.[16, 42] The regression model estimates the hazard of initiation for individual $i$ in period $t$ ($y_{it}$) as a function of real cigarette prices ($price_t$) and a matrix of explanatory variables ($\boldsymbol{X}_i$), which include socio-demographics (age, gender, parents' and friends' smoking status, income (pocket money) and respondents' perception on quitting smoking). Gender takes the value of 1 for female and 0 for male. Smoking status of the respondent's parents and friends takes the value of 1 if at least one of the parents or friends smokes, and 0 if none of the parents or friends smoke. The respondents' income (pocket money) is derived from the question: "During an average week, how much money do you have that you can spend on yourself, however you want?". The base is 'I usually don't have', and the six other categories are 'Less than GMD25', 'GMD25-GMD50', 'GMD51-GMD100', 'GMD101-GMD150', 'GMD151-GMD200' and 'Above GMD200'. Respondents' perception on quitting smoking is derived from the GYTS question: "Once someone has started smoking tobacco, do you think it would be difficult for them to quit?" The variable is coded as 1 for "Yes" and 0 for "No". The dependent variable, i.e. cigarette smoking experimentation, is extracted from the GYTS data.[34]

The regression model is specified as:

$$y_{it} = Pr\ (initiate|no\ prior\ smoking)$$



For peer review only - http://bmjopen.bmj.com/site/about/guidelines.xhtml

$$= \beta_1 price_t + \beta_2 \boldsymbol{X}_{it} + \varepsilon_{it} \text{ ........ (1)}$$

We tested the robustness of the results by incorporating a discrete-time split population survival model, which relaxes the assumption that all individuals will eventually smoke. It first estimates each individual's probability of ever experiencing a smoking transition, then weights the hazard function by this probability. The contribution of individual *i* to the log-likelihood function of smoking experimentation is:

$$d_i = ln\{\Pr(ever\ initiate) * f(t \mid t > 0)\} + (1 - d_i) * ln \{Pr(ever\ initiate) + Pr(ever\ initiate) * f(t|t = 0)\} \text{ ......... (2)}$$

where $d_i$ is a binary indicator for experimenting with smoking at some point during the period of observation, *t* is the time of experimentation measured in number of years since age eight, and *f*(*t*) is its probability density function.

As in Vellios and Van Walbeek[38], we used Stata's *spsurv* command for the split population estimation model. The model has been used in numerous studies.[38, 43, 44] The *spsurv* command uses a complementary log-log specification, which reports hazard ratios (not odds ratios) and unlike the logit specification, the response curve is asymmetric.[42] The odds ratios are calculated by taking the antilog of the coefficient. The hazard rate is calculated as (OR / (1 + OR) where OR is the odds ratio. All analyses were done with Stata v.15.

**Patients and public involvement**

Patients and the public were not involved in this study.



For peer review only - http://bmjopen.bmj.com/site/about/guidelines.xhtml

## Results

The regression results are shown in Table 2. The estimated odds and hazard ratios are in exponentiated form and are broadly similar. The hazard ratios are indicated in the table but are not discussed in this section.

**Table 2: Regression results: logistic and split population survival models**

| | Logistic regression model | | Split population model |
|---|---|---|---|
| | Coefficients | Odds ratios | Hazard ratios |
| | (1) | (2) | (3) |
| Price elasticity of experimentation | -0.680** | 0.507** | 0.524*** |
| | (0.302) | (0.153) | (0.131) |
| Parent(s) smoke(s) | 0.371*** | 1.449*** | 1.474*** |
| | (0.082) | (0.120) | (0.093) |
| Friend(s) smoke(s) | 0.482*** | 1.620*** | 1.828*** |
| | (0.073) | (0.119) | (0.102) |
| Difficulty of quitting | 0.055 | 1.056 | 1.114* |
| | (0.069) | (0.073) | (0.062) |
| Teachers smoke in school buildings | 0.176** | 1.192** | 1.205*** |
| | (0.073) | (0.087) | (0.068) |
| Usually do not have pocket money | 0.000 | 1.000 | 1.000 |
| Less than GMD25 | 0.083 | 1.087 | 1.113 |
| | (0.127) | (0.138) | (0.109) |
| GMD25-GMD50 | 0.067 | 1.070 | 1.181* |
| | (0.125) | (0.134) | (0.114) |
| GMD51-GMD100 | 0.334** | 1.397** | 1.400*** |
| | (0.141) | (0.197) | (0.158) |
| GMD101-GMD150 | 0.080 | 1.084 | 1.224 |
| | (0.190) | (0.206) | (0.190) |
| GMD151-GMD200 | 0.246 | 1.279 | 1.396* |
| | (0.227) | (0.290) | (0.261) |
| Above GMD200 | 0.411** | 1.508** | 1.505*** |
| | (0.181) | (0.273) | (0.224) |
| Female | -1.403*** | 0.246*** | 1.159*** |
| | (0.083) | (0.020) | (0.016) |
| Age | 0.165*** | 1.179*** | 1.159*** |
| | (0.020) | (0.024) | (0.016) |
| Constant | -3.426*** | 0.033*** | 0.028*** |
| | (0.892) | (0.029) | (0.021) |



For peer review only - http://bmjopen.bmj.com/site/about/guidelines.xhtml

| | | | |
|---|---|---|---|
| Observations | 77,640 | 77,640 | 77,640 |

Standard errors in parentheses
*** p<0.01, ** p<0.05, * p<0.10

The results show that a 1% increase in the price of cigarettes reduces the probability to experiment with smoking among children by 0.7% (95% CI: -1.1 to -0.4) (as indicated in column 1).

Children with at least one smoking parent are more likely to have ever smoked a cigarette than those whose parents do not smoke (OR = 1.4, 95% CI: 1.2 to 1.7). Also, children with friends who smoke are more likely to have ever experimented with smoking a cigarette than those whose friends do not smoke (OR = 1.6, 95% CI: 0.4 to 1.9).

Children who see their teachers smoking inside school buildings are more likely to have ever experimented with smoking a cigarette than those who do not see teachers smoking inside school buildings (OR = 1.2, 95% CI: 1.0 to 1.4). Income (i.e., having access to pocket money) did not have a clear effect, although there is some suggestion that children who receive pocket money are more likely to experiment with smoking than children who do not receive pocket money.[45]

Female youths are less likely to have ever smoked a cigarette than male youths (OR = 0.2, 95% CI: 0.2 to 0.3). The results also show that a one-year increase in age increases the probability to experiment with smoking (OR = 1.2, 95% CI:1.1 to 1.2). Figure 2 show the smoking initiation hazard rates by age and gender. The hazard rates are derived from the regression analyses.

*Insert Figure 2 here*

Figure 2 shows a positive correlation between age and the probability to experiment with smoking cigarettes among the youth in The Gambia. The probability to experiment with smoking is higher among boys than among girls.



For peer review only - http://bmjopen.bmj.com/site/about/guidelines.xhtml

## Discussion

The price elasticity of demand for smoking experimentation is estimated to be -0.7 among children in The Gambia. This implies that a 10% rise in the price of cigarettes is associated with a 7% reduction in the probability to experiment with smoking among the Gambian children. Increasing excise taxes could be a highly effective tobacco-control tool in The Gambia. The excise tax typically increases the retail price of cigarettes, thereby reducing the propensity to experiment with smoking.[1, 16] Considering that the current tax burden of 46.3% is well below the 75% target recommended by the World Health Organisation,[46] there is room for the government to further raise the excise tax on tobacco. Raising tobacco tax is a 'win-win' policy, as it generates extra revenue for the government while discouraging tobacco consumption through higher prices, thereby improving public health.[29]

The study established that children with smoking friends were more likely to have ever smoked a cigarette than those whose friends do not smoke. Similarly, children from households with at least one parent who currently smokes were more likely to have ever smoked a cigarette. This could be because, as children are more exposed to smoking (especially by parents) they would regard smoking as normal. Also, if the parents smoke, access to cigarettes is easier, than if the parents do not smoke. The results from this study are in line with the findings of Jallow et al[47] who found that children in smoking families were more likely to smoke than those in non-smoking families. We also found that children are more likely to experiment with cigarette smoking if they see their teachers smoking in school buildings. If smoke-free regulations are effectively implemented, it would presumably reduce smoking in school buildings, and thus reducing youth smoking. Nevertheless, our study found that peer influence was more important in experimenting with smoking than both parental and teachers' influence. A systematic review of studies conducted



For peer review only - http://bmjopen.bmj.com/site/about/guidelines.xhtml

among high school and university students in Ethiopia and other studies in the US found that peer pressure had a significant influence on young peoples' initiation and smoking of cigarettes.[33, 48] Increases in the price will have a direct and indirect influence on smoking. The direct influence will be through the price elasticity of demand. The indirect influence will be through the peers. If the price increases, the peers will smoke less (or not at all), thus decreasing the peer pressure.

The Gambian youths start smoking as early as at age eight, a result similar to a previously published study.[26] The likelihood of smoking increases exponentially as age increases. As in most other countries, the probability of smoking is higher among boys than among girls. This is mirrored by the smoking prevalence in the general population, where smoking prevalence among women is very low (about 1%) compared to men (32.1%).[26] However, the smoking rate among girls is substantially higher than among adult women, suggesting that female smoking prevalence may increase sharply in future, as girls step into adulthood. This would be detrimental to public health. The government should implement policies, including an increase in the excise tax, that will prevent an increase in smoking experimentation.

## Limitation of the study

Although this study provides useful information for devising suitable tobacco-tax policy measures, there are some limitations to consider. For instance, the study relied on individuals' own memory regarding past smoking behaviour. As such, the dependent variable could be subject to measurement error with an unknown bias. However, this challenge is more prevalent among adult respondents.[16, 19] Considering that our sample was composed of children, with relatively short smoking histories, we believe that our findings are robust. In addition, the study relied on a pseudo-panel where several individual demographic and socio-economic characteristics were fixed at the time of the survey. To the extent that some of these socio-economic variables have changed in the



For peer review only - http://bmjopen.bmj.com/site/about/guidelines.xhtml

years prior to the survey, this could have biased the results. Also, considering that the 2017 GYTS is a cross-sectional survey among students in junior secondary schools, the data may not be a good representation of Gambian youth, e.g. those in senior secondary schools (Grades 10-12) and school dropouts. We do not have information on what happens beyond this age group, because the data are not collected. In addition, some important information such as the respondents' region, race, and religion were not collected. Had these variables been available, they could have been used in the regression analysis.

## Conclusion

We found the price elasticity of demand for smoking experimentation to be -0.7 among children who have smoked at least once or twice in their lives. Our results indicate that an increase in tobacco excise taxes can play an effective role in discouraging children from experimenting with smoking. Considering the current relatively low excise tax burden, the government should consider increasing the excise tax burden on tobacco products in line with the recommendations of the World Health Organisation.[32]

**Acknowledgements:** Special thanks to Nicole Vellios who reviewed earlier drafts.

**Contributors:** CD and MKB conceptualised the study and conducted the data analysis. CD, MKB and CB wrote the first draft of the manuscript. CvW, ZG and UD'A contributed to the drafting and revision of the manuscript.

**Funding:** This study was primarily supported by the UK Research and Innovation (UKRI) with funding from the Global Challenges Research Fund (MR/P027946/2), as part of the Tobacco Control Capacity Programme (TCCP). The TCCP is a programme of capacity development and



For peer review only - http://bmjopen.bmj.com/site/about/guidelines.xhtml

research co-ordinated by the University of Edinburgh, Scotland and involves 15 partner institutions from Africa, South Asia and the United Kingdom. The study was also supported by the South African Medical Research Council (grant no: 23108).

**Competing interests:** None.

**Patient consent for publication:** Not required.

**Ethics approval:** N/A. The data is publicly available.

**Data sharing statement:** Data are publicly available on: https://nccd.cdc.gov/GTSSDataSurveyResources/Ancillary/DataReports.aspx?CAID=2.

## References

[1] National Cancer Institute and World Health Organization. The Economics of Tobacco and Tobacco Control: National Cancer Institute Tobacco Control Monograph 21 NIH Publication No 16-CA-8029A: Bethesda, MD: U.S. Department of Health and Human Services, National Institutes of Health, National Cancer Institute; and Geneva, CH: World Health Organization; 2016. Available from: https://cancercontrol.cancer.gov/sites/default/files/2020-06/m21_complete.pdf.

[2] World Health Organization. Global Health Observatory data: prevalence of tobacco smoking. Web address: http://wwwwhoint/gho/tobacco/use/en/, Accessed date. 2020;28.

[3] Bilano V, Gilmour S, Moffiet T, d'Espaignet ET, Stevens GA, Commar A, et al. Global trends and projections for tobacco use, 1990–2025: an analysis of smoking indicators from the WHO Comprehensive Information Systems for Tobacco Control. The Lancet. 2015;385(9972):966-76.



For peer review only - http://bmjopen.bmj.com/site/about/guidelines.xhtml

[4] World health Organization. Tobacco Factsheet 2020 [Available from: https://www.who.int/en/news-room/fact-sheets/detail/tobacco [Accessed 02 March 2021].

[5] Forouzanfar MH, Afshin A, Alexander LT, Anderson HR, Bhutta ZA, Biryukov S, et al. Global, regional, and national comparative risk assessment of 79 behavioural, environmental and occupational, and metabolic risks or clusters of risks, 1990–2015: a systematic analysis for the Global Burden of Disease Study 2015. The lancet. 2016;388(10053):1659-724.

[6] Blecher EH, Ross H. Tobacco use in Africa: tobacco control through prevention. American Cancer Society. 2015.

[7] Chandora R, Song Y, Chaussard M, Palipudi KM, Lee KA, Ramanandraibe N, et al. Youth access to cigarettes in six sub-Saharan African countries. Preventive Medicine. 2016;91:S23-S7.

[8] Organization WH. WHO report on the global tobacco epidemic, 2013: enforcing bans on tobacco advertising, promotion and sponsorship: World Health Organization; 2013.

[9] World Health Organization. WHO global report on trends in prevalence of tobacco smoking 2000-2025: World Health Organization; 2018.

[10] Sargent JD, Gabrielli J, Budney A, Soneji S, Wills TA. Adolescent smoking experimentation as a predictor of daily cigarette smoking. Drug and Alcohol Dependence. 2017;175:55-9.

[11] Friedman AS, Buckell J, Sindelar JL. Patterns of youth cigarette experimentation and onset of habitual smoking. American journal of preventive medicine. 2019;56(6):803-10.

[12] Ho L-M, Schafferer C, Lee J-M, Yeh C-Y, Hsieh C-J. The effect of cigarette price increases on cigarette consumption, tax revenue, and smoking-related death in Africa from 1999 to 2013. International journal of public health. 2017;62(8):899-909.



For peer review only

For peer review only - http://bmjopen.bmj.com/site/about/guidelines.xhtml

[13] Boachie MK, Immurana M, Agyemang JK, Ross H. Cigarette prices and smoking experimentation in Sierra Leone: an exploratory study. Tobacco Use Insights. 2022;15:1179173X221078189.

[14] Mukong AK, Tingum EN. The demand for cigarettes: New evidence from South Africa. Development Southern Africa. 2020;37(1):40-54.

[15] Kidane A, Mduma J, Naho A, Ngeh ET, Hu T-w. The demand for cigarettes in tanzania and implications for tobacco taxation policy. Advances in economics and business. 2015;3(10):428.

[16] Dauchy E, Ross H. The effect of price and tax policies on the decision to smoke among men in Kenya. Addiction. 2019;114(7):1249-63.

[17] Stoklosa M, Goma F, Nargis N, Drope J, Chelwa G, Chisha Z, et al. Price, tax and tobacco product substitution in Zambia: findings from the ITC Zambia Surveys. Tobacco Control. 2019;28(Suppl 1):s45-s52.

[18] Chelwa G, Van Walbeek C. Does cigarette demand respond to price increases in Uganda? Price elasticity estimates using the Uganda National Panel Survey and Deaton's method. BMJ open. 2019;9(3):e026150.

[19] Asare S, Stoklosa M, Drope J, Larsen A. Effects of Prices on Youth Cigarette Smoking and Tobacco Use Initiation in Ghana and Nigeria. International journal of environmental research and public health. 2019;16(17):3114.

[20] United Nations Development Programme. Human Development Insight 2022 [Available from: https://hdr.undp.org/data-center/country-insights#/ranks.



For peer review only - http://bmjopen.bmj.com/site/about/guidelines.xhtml

[21] World Bank. GDP per capita (current US$) - Lower middle income, Gambia, The. 2022 [Available from: https://data.worldbank.org/indicator/NY.GDP.PCAP.CD?locations=XN-GM.

[22] United Nations. United Nations Treat Collections. 2022 [Available from: https://treaties.un.org/pages/ViewDetails.aspx?src=TREATY&mtdsg_no=IX-4&chapter=9&clang=_en.

[23] World Health Organization. Legislation by country: Gambia 2022 [Available from: https://www.tobaccocontrollaws.org/legislation/country/gambia/summary#:~:text=The%20law%20prohibits%20the%20sale,worship%2C%20and%20other%20specified%20locations.

[24] Cham B, Mdege ND, Bauld L, Britton J, D'alessandro U. Exposure to Second-Hand Smoke in Public Places and Barriers to the Implementation of Smoke-Free Regulations in The Gambia: A Population-Based Survey. International journal of environmental research and public health. 2021;18(12):6263.

[25] Cham B, Scholes S, Ng Fat L, Badjie O, Mindell JS. Burden of hypertension in The Gambia: evidence from a national World Health Organization (WHO) STEP survey. International journal of epidemiology. 2018;47(3):860-71.

[26] Cham B, Scholes S, Groce NE, Mindell JS. Prevalence and predictors of smoking among Gambian men: a cross-sectional national WHO STEP survey. International journal of environmental research and public health. 2019;16(23):4719.

[27] Jallow IK, Britton J, Langley T. Prevalence and determinants of tobacco use among young people in The Gambia. BMJ global health. 2017;2(4).

[28] Manneh E, Gambia. A Global Youth Tobacco Survey (GYTS) Country Report 2008. Government of The Gambia: Banjul, The Gambia. 2008.



For peer review only - http://bmjopen.bmj.com/site/about/guidelines.xhtml

[29] Nargis N, Manneh Y, Krubally B, Jobe B, Ouma AEO, Tcha-Kondor N, et al. How effective has tobacco tax increase been in the Gambia? A case study of tobacco control. BMJ open. 2016;6(8):e010413.

[30] The Gambia Bureau of Statistics. 2020.

[31] World Health Organization. WHO report on the global tobacco epidemic 2019: Offer help to quit tobacco use. Geneva: World Health Organization; 2019.

[32] World Health Organization. Taxation 2020 [Available from: https://www.euro.who.int/en/health-topics/disease-prevention/tobacco/publications/key-policy-documents/who-framework-convention-on-tobacco-control-who-fctc/key-areas-of-tobacco-control-policy/taxation Access: 20 July 2020.

[33] IARC. IARC handbooks of cancer prevention, tobacco control, vol. 14: effectiveness of tax and price policies for tobacco control. Lyon, France. 2011.

[dataset] [34] Centers for Disease Control and Prevention. Global Tobacco Surveillance System Data. 2021 [Available from: https://nccd.cdc.gov/GTSSDataSurveyResources/Ancillary/DataReports.aspx?CAID=2 [Accessed: 23 January 2021].

[35] Cadmus EO, Ayo-Yusuf OA. The effect of smokeless tobacco use and exposure to cigarette promotions on smoking intention among youths in Ghana. Cogent Medicine. 2018;5(1):1531459.

[36] Boachie MK, Immurana M, Tingum EN, Mdege ND, Ross H. Effect of relative income price on smoking initiation among adolescents in Ghana: evidence from pseudo-longitudinal data. BMJ open. 2022;12(3):e054367.



For peer review only - http://bmjopen.bmj.com/site/about/guidelines.xhtml

[37] Schober P, Vetter TR. Survival analysis and interpretation of time-to-event data: the tortoise and the hare. Anesthesia and analgesia. 2018;127(3):792.

[38] Vellios N, van Walbeek C. Determinants of regular smoking onset in South Africa using duration analysis. BMJ open. 2016;6(7).

[39] Guindon GE, Paraje GR, Chávez R. Prices, inflation, and smoking onset: the case of Argentina. Economic Inquiry. 2018;56(1):424-45.

[40] Marti J. The impact of tobacco control expenditures on smoking initiation and cessation. Health economics. 2014;23(12):1397-410.

[41] Madden D. Tobacco taxes and starting and quitting smoking: does the effect differ by education? Applied Economics. 2007;39(5):613-27.

[42] Lambert PC. Modeling of the cure fraction in survival studies. The Stata Journal. 2007;7(3):351-75.

[43] Lillard DR, Molloy E, Sfekas A. Smoking initiation and the iron law of demand. Journal of health economics. 2013;32(1):114-27.

[44] Kidd MP, Hopkins S. The hazards of starting and quitting smoking: some Australian evidence. Economic Record. 2004;80(249):177-92.

[45] Cui Y, Forget EL, Zhu Y, Torabi M, Oguzoglu U. The effects of cigarette price and the amount of pocket money on youth smoking initiation and intensity in Canada. Canadian Journal of Public Health. 2019;110(1):93-102.

[46] World Health Organization. Taxation 2021 [Available from: https://www.euro.who.int/en/health-topics/disease-prevention/tobacco/publications/key-policy-documents/who-framework-convention-on-tobacco-control-who-fctc/key-areas-of-tobacco-control-policy/taxation.

For peer review only



For peer review only - http://bmjopen.bmj.com/site/about/guidelines.xhtml

[47] Jallow IK, Britton J, Langley T. Prevalence and determinants of susceptibility to tobacco smoking among students in the Gambia. Nicotine and Tobacco Research. 2019;21(8):1113-21.

[48] Leshargie CT, Alebel A, Kibret GD, Birhanu MY, Mulugeta H, Malloy P, et al. The impact of peer pressure on cigarette smoking among high school and university students in Ethiopia: A systemic review and meta-analysis. PloS one. 2019;14(10):e0222572.

**Figure 1: Average real price, 2008 – 2017**

**Figure 2: Smoking hazard rates for smoking experimentation**

For peer review only

**Figure 1: Average real price, 2008 – 2017**

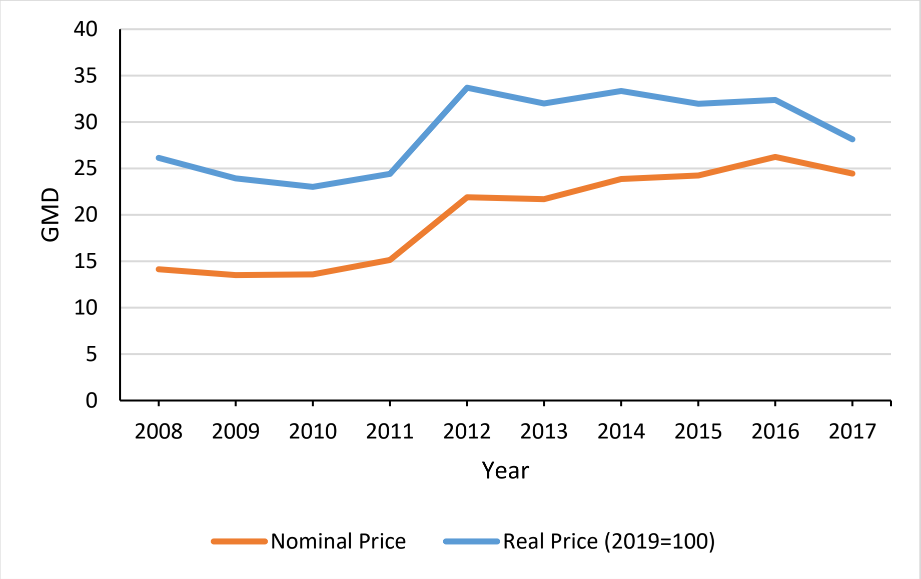


**Source:** The Gambia Bureau of Statistics

**Figure 2: Smoking hazard rates for smoking experimentation**

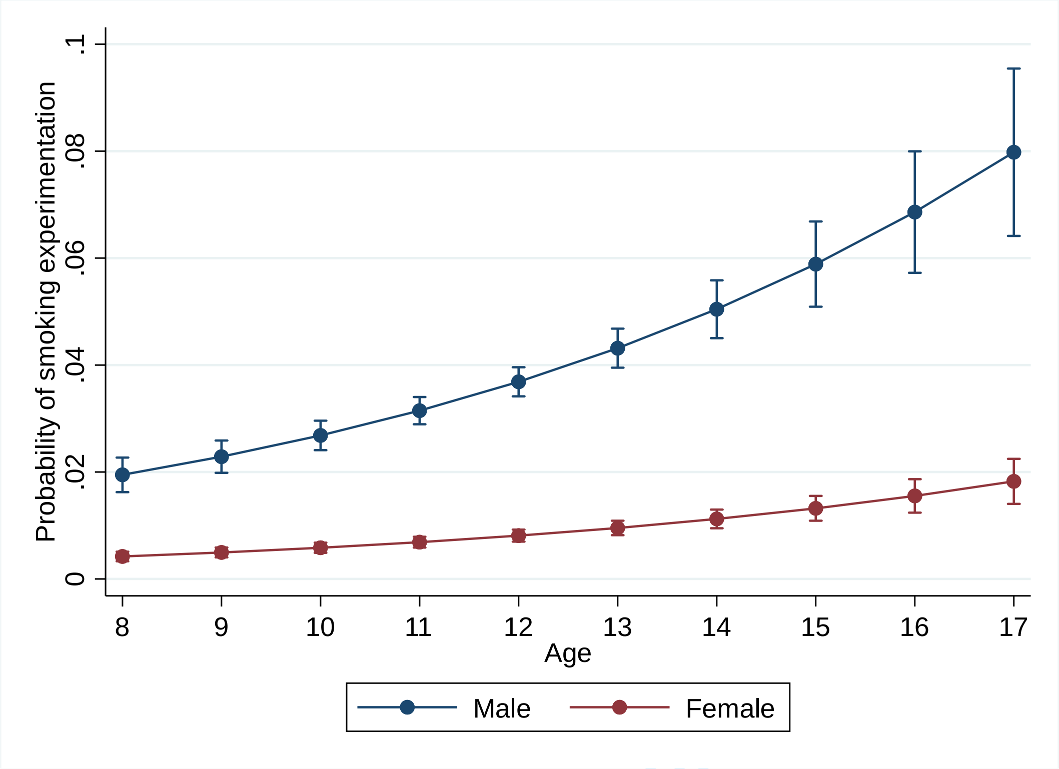


For peer review only

STROBE Statement—Checklist of items that should be included in reports of ***cross-sectional studies***

| | Item No | Recommendation | Page No |
|---|---|---|---|
| **Title and abstract** | 1 | (*a*) Indicate the study's design with a commonly used term in the title or the abstract | 2 |
| | | (*b*) Provide in the abstract an informative and balanced summary of what was done and what was found | 3 |
| **Introduction** | | | |
| Background/rationale | 2 | Explain the scientific background and rationale for the investigation being reported | 4-6 |
| Objectives | 3 | State specific objectives, including any prespecified hypotheses | 6 |
| **Methods** | | | |
| Study design | 4 | Present key elements of study design early in the paper | 7 |
| Setting | 5 | Describe the setting, locations, and relevant dates, including periods of recruitment, exposure, follow-up, and data collection | 7-8 |
| Participants | 6 | (*a*) Give the eligibility criteria, and the sources and methods of selection of participants | 8 |
| Variables | 7 | Clearly define all outcomes, exposures, predictors, potential confounders, and effect modifiers. Give diagnostic criteria, if applicable | 8-9 |
| Data sources/ measurement | 8* | For each variable of interest, give sources of data and details of methods of assessment (measurement). Describe comparability of assessment methods if there is more than one group | 8-9 |
| Bias | 9 | Describe any efforts to address potential sources of bias | 10 |
| Study size | 10 | Explain how the study size was arrived at | 8 |
| Quantitative variables | 11 | Explain how quantitative variables were handled in the analyses. If applicable, describe which groupings were chosen and why | 9 |
| Statistical methods | 12 | (*a*) Describe all statistical methods, including those used to control for confounding | 9-10 |
| | | (*b*) Describe any methods used to examine subgroups and interactions | 9-10 |
| | | (*c*) Explain how missing data were addressed | 8 |
| | | (*d*) If applicable, describe analytical methods taking account of sampling strategy | 7-8 |
| | | (*e*) Describe any sensitivity analyses | 9 |
| **Results** | | | |
| Participants | 13* | (a) Report numbers of individuals at each stage of study—eg numbers potentially eligible, examined for eligibility, confirmed eligible, included in the study, completing follow-up, and analysed | 8 |
| | | (b) Give reasons for non-participation at each stage | |
| | | (c) Consider use of a flow diagram | |
| Descriptive data | 14* | (a) Give characteristics of study participants (eg demographic, clinical, social) and information on exposures and potential confounders | 7 |
| | | (b) Indicate number of participants with missing data for each variable of interest | |
| Outcome data | 15* | Report numbers of outcome events or summary measures | |
| Main results | 16 | (*a*) Give unadjusted estimates and, if applicable, confounder-adjusted estimates and their precision (eg, 95% confidence interval). Make clear which confounders were adjusted for and why they were included | 11-12 |



For peer review only - http://bmjopen.bmj.com/site/about/guidelines.xhtml

| | | | |
|---|---|---|---|
| | | (*b*) Report category boundaries when continuous variables were categorized | |
| | | (*c*) If relevant, consider translating estimates of relative risk into absolute risk for a meaningful time period | |
| Other analyses | 17 | Report other analyses done—eg analyses of subgroups and interactions, and sensitivity analyses | |
| **Discussion** | | | |
| Key results | 18 | Summarise key results with reference to study objectives | 10-12 |
| Limitations | 19 | Discuss limitations of the study, taking into account sources of potential bias or imprecision. Discuss both direction and magnitude of any potential bias | 14 |
| Interpretation | 20 | Give a cautious overall interpretation of results considering objectives, limitations, multiplicity of analyses, results from similar studies, and other relevant evidence | 12-13 |
| Generalisability | 21 | Discuss the generalisability (external validity) of the study results | 14 |
| **Other information** | | | |
| Funding | 22 | Give the source of funding and the role of the funders for the present study and, if applicable, for the original study on which the present article is based | 15 |

*Give information separately for exposed and unexposed groups.

**Note:** An Explanation and Elaboration article discusses each checklist item and gives methodological background and published examples of transparent reporting. The STROBE checklist is best used in conjunction with this article (freely available on the Web sites of PLoS Medicine at http://www.plosmedicine.org/, Annals of Internal Medicine at http://www.annals.org/, and Epidemiology at http://www.epidem.com/). Information on the STROBE Initiative is available at www.strobe-statement.org.

For peer review only
